# Deep transfer learning of structural magnetic resonance imaging fused with blood parameters improves brain age prediction

**DOI:** 10.1002/hbm.25748

**Published:** 2021-12-16

**Authors:** Bingyu Ren, Yingtong Wu, Liumei Huang, Zhiguo Zhang, Bingsheng Huang, Huajie Zhang, Jinting Ma, Bing Li, Xukun Liu, Guangyao Wu, Jian Zhang, Liming Shen, Qiong Liu, Jiazuan Ni

**Affiliations:** ^1^ Shenzhen Key Laboratory of Marine Biotechnology and Ecology, College of Life Sciences and Oceanography Shenzhen University Shenzhen China; ^2^ Medical AI Lab, School of Biomedical Engineering, Health Science Center Shenzhen University Shenzhen China; ^3^ MIND Lab, School of Biomedical Engineering, Health Science Center Shenzhen University Shenzhen China; ^4^ Shenzhen‐Hong Kong Institute of Brain Science‐Shenzhen Fundamental Research Institutions Shenzhen China; ^5^ Radiology Department Shenzhen University General Hospital and Shenzhen University Clinical Medical Academy, Shenzhen University Shenzhen China; ^6^ Health Science Center Shenzhen University Shenzhen China; ^7^ Shenzhen Bay Laboratory Shenzhen China

**Keywords:** blood biochemical indicators, brain age, deep transfer learning, dementia‐associated biomarkers, magnetic resonance imaging, multimodal data fusion

## Abstract

Machine learning has been applied to neuroimaging data for estimating brain age and capturing early cognitive impairment in neurodegenerative diseases. Blood parameters like neurofilament light chain are associated with aging. In order to improve brain age predictive accuracy, we constructed a model based on both brain structural magnetic resonance imaging (sMRI) and blood parameters. Healthy subjects (*n* = 93; 37 males; aged 50–85 years) were recruited. A deep learning network was firstly pretrained on a large set of MRI scans (*n* = 1,481; 659 males; aged 50–85 years) downloaded from multiple open‐source datasets, to provide weights on our recruited dataset. Evaluating the network on the recruited dataset resulted in mean absolute error (MAE) of 4.91 years and a high correlation (*r* = .67, *p* <.001) against chronological age. The sMRI data were then combined with five blood biochemical indicators including GLU, TG, TC, ApoA1 and ApoB, and 9 dementia‐associated biomarkers including ApoE genotype, HCY, NFL, TREM2, Aβ40, Aβ42, T‐tau, TIMP1, and VLDLR to construct a bilinear fusion model, which achieved a more accurate prediction of brain age (MAE, 3.96 years; *r* = .76, *p* <.001). Notably, the fusion model achieved better improvement in the group of older subjects (70–85 years). Extracted attention maps of the network showed that amygdala, pallidum, and olfactory were effective for age estimation. Mediation analysis further showed that brain structural features and blood parameters provided independent and significant impact. The constructed age prediction model may have promising potential in evaluation of brain health based on MRI and blood parameters.

## Graphical abstract

Deep transfer learning of structural MRI fused with blood parameters improves brain age prediction in healthy elder subjects.

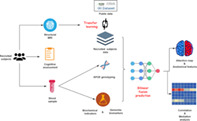

## 1 INTRODUCTION

Numerous studies have demonstrated that the morphology of human brain changes during aging process (Oschwald et al., 2019). Furthermore, neurodegenerative diseases, such as Alzheimer's disease (AD), have been reported to show accelerated brain aging and atrophy (Gellersen et al., 2017). Higher predicted brain age has been found to be associated with many neuropsychiatric disorders, including not only neurodegeneration such as mild cognitive impairment (MCI) and AD, but also traumatic brain injury, schizophrenia, epilepsy, and Down's syndrome (Cole & Franke, 2017). The “age gap” between the predicted brain age and the chronological age is considered as a potential biomarker for evaluating brain health (Bashyam et al., 2020).

The structural magnetic resonance imaging (sMRI) scans provide anatomical information of the brain regions, thus capturing the age‐related brain changes (Grajauskas et al., 2019). Brain age prediction models based on sMRI and machine learning show promising prospects in studying brain aging and identifying early‐stage neurodegeneration (Sajedi & Pardakhti, 2019). In order to further improve the age predictive accuracy, fusion of multimodal information has been regarded as a promising strategy. Previous attempts mainly focused on the combination of imaging data in different sequences, for instance, sMRI, diffusion MRI, and functional MRI (Liem et al., 2017; Niu, Zhang, Kounios, & Liang, 2020; Rokicki et al., 2021). However, brain age and the rate of cognitive decline in middle‐to‐old‐age population are not only related to their brain structure, but also to factors like neurochemical parameters that cannot be obtained from neuroimaging directly (Habes et al., 2021). Meanwhile, a key observation in neuroimaging‐based brain age prediction is that the predicted age is higher than the chronological age for younger subjects and lower for older subjects (Feng, Lipton, Yang, Small, & Provenzano, 2020; Sagers, Melas‐Kyriazi, Patel, & Manrai, 2020). One possibility is that human heterogeneity arose from genetic differences or subtle effects of the environment, such as a brain injury or cerebral infection, leads to changes in brain structure (Cole & Franke, 2017).

To adjust the brain age error caused by nonaging‐related changes in brain structure, blood parameters may be a solution. Blood biochemical indicators and dementia‐associated biomarkers extracted from blood are reported to change with aging. Biochemical indicators like the total cholesterol (TC) and triglycerides (TG) have been shown to change with aging (Kreisberg & Kasim, 1987), while the decline in renal functions, nutritional deficiencies and deficiencies of homocysteine (HCY) remethylation cause elevation of HCY with advancing age (Ostrakhovitch & Tabibzadeh, 2019). Also, several reports have discovered positive correlations between chronological age and the dementia‐associated biomarkers including plasma T‐tau (Nakamura et al., 2018), amyloid‐beta (Aβ) 42 levels (Lue et al., 2019), and neurofilament light chain (NFL; Khalil et al., 2020).

Recently, there has been an emerging trend to integrate imaging and biomarker data. The blood biochemical indicators and dementia‐associated biomarkers are easily‐obtained circulating markers that represent the health state of the whole body, including the brain. Ly et al. (2020) tried combining amyloid status with sMRI to improve brain age prediction. They pointed out that if amyloid was not taken into consideration, it might lead to a bias in predicting brain age. But given the diversity of potential biomarkers associated with aging or neurodegenerative diseases, it was not comprehensive to consider amyloid only.

In this work, we aimed to construct a brain age prediction model applicable in the Chinese elderly population, and hypothesized that sMRI and the blood parameters provide nonoverlapping information which can improve predictive accuracy in brain age prediction. Age‐appropriate healthy participants were recruited and their brain sMRI and 14 blood parameters were collected. To be specific, blood parameters are five clinically feasible biochemical indicators including glucose (GLU), TG, TC, apolipoprotein A1 (ApoA1), and apolipoprotein B (ApoB), and nine dementia biomarkers that represent the subjects' brain health states including ApoE genotype, HCY, NFL, Triggering Receptor Expressed on Myeloid cells 2 (TREM2), Aβ40, Aβ42, T‐tau, Tissue Inhibitor of Metalloproteinases 1 (TIMP1), and Very Low Density Lipoprotein Receptor (VLDLR). A model was pretrained on a large public dataset of brain sMRI and was applied to our recruited dataset by using deep transfer learning. Then the recruited subjects' sMRI and blood parameters data were incorporated by using a multimodal linear fusion approach for better predictive performance. The interpretability of multiple features was described by attention maps, mediation analysis and principal component analysis (PCA).

## 2 MATERIALS AND METHODS

### 2.1 Participants

Thirty‐seven male and fifty‐six female Chinese volunteers (over 50 years old) were recruited from Shenzhen University General Hospital. All participants were mentally healthy individuals. The experiments were performed with the written informed consent of all participants. This retrospective study protocol was approved by the Institutional Review Board of Shenzhen University General Hospital. The procedures conducted in this study were adherent to the principles of the Declaration of Helsinki. Interviews of medical history were conducted, to rule out cases of traumatic brain injuries and clinically diagnosed neurological disease. The Mini‐Mental State Examination (MMSE) and Montreal Cognitive Assessment (MoCA) Tests were applied to assess the cognitive state of the participants after blood glucose tests and collection of their blood samples. The whole dataset contained 93 subjects from which 16 were excluded due to the following reasons: (1) six were excluded because they had infective hepatitis, contraindications to MRI such as metallic implants, or claustrophobia. Their MRI scans or blood parameters cannot be obtained, (2) seven were excluded because they suffered from neurological disorders or had structural lesions, (3) three were excluded because their MMSE and MoCA scores were both below 25 (Nair, Ramaswamy, Kan, & Nair, 2019; Versijpt et al., 2017). Patients with neurological diseases including Parkinson's disease, AD and MCI, and patients with cerebellar atrophy, intracranial hemorrhage and cerebral infarction, were all defined as having structural brain lesions.

### 2.2 Data acquisition and preprocessing

#### 2.2.1 Data acquisition

The sMRI scans were performed on a 3T MRI scanner (Discovery MR750, GE Healthcare, Milwaukee, WI) with an eight‐channel phased array head and neck coil. A high‐resolution three‐dimensional T1‐weighted structural imaging was performed by using a brain volume (BRAVO) sequence with the following parameters: repetition time (TR) = 6.7 ms; echo time (TE) = 2.9 ms; flip angle = 12°; acquisition matrix = 256 × 256; bandwidth = 31.25; number of excitations = 1 and slice thickness = 1 mm with a 0‐mm gap; total slices = 180.

The sMRI data for pretraining the deep learning network were collected from three public databases including Alzheimer's Disease Neuroimaging Initiative (ADNI), Information eXtraction from Images (IXI), and the Open Access Series of Imaging Studies (OASIS). The subjects we collected were between 50 and 85 years of age and cognitively normal. The detailed information of the downloaded dataset and our recruited dataset was provided in Tables 1 and 2. See Figure S2 for open‐source dataset selection strategies.

**TABLE 1:** Group demographics of our recruited subjects

	50–60	60–70	70–85	Group comparison	Post hoc
*n*	33	30	14	N/A	N/A
Sex	20 females, 13 males	18 females, 12 males	9 females, 5 males	*χ* ^2^ = 0.078, *p* = 0.962^a^	N/A
MMSE score	28.9 ± 1.2 (26–30)	28.7 ± 1.3 (26–30)	28.0 ± 1.4 (25–30)	*F* = 2.102, *p* = .129^b^	50–60 vs. 60–70, *p* >.999 50–60 vs. 70–85, *p* = .133 60–70 vs. 70–85, *p* = .404
MoCA‐B score	28.0 ± 2.1 (20–30)	26.8 ± 1.9 (20–29)	26.0 ± 3.1 (18–30)	*F* = 3.248, *p* = .045^b^	50–60 vs. 60–70, *p* = .124 50–60 vs. 70–85, *p* = .106 60–70 vs. 70–85, *p* >.999
APOE ε4 carrier	9 carriers, 24 noncarriers	3 carriers, 27 noncarriers	0 carriers, 14 noncarriers	*χ* ^2^ = 6.723, *p = .035^a^	N/A

*Note*: The MMSE and MoCA‐B scores were given as mean ± standard deviation (range).

^a^ Chi‐squared test.

^b^ One‐way ANOVA.

**TABLE 2:** Group demographics of the subjects downloaded from the public dataset

	50–60	60–70	70–80	80–85	Group comparison
ADNI	0	84	441	158	N/A
IXI	99	118	49	6	N/A
OASIS	150	376	0	0	N/A
Total	249	578	490	164	N/A
Sex	150 (60.2%) females, 99 (39.7%) males	317 (54.8%) females, 261 (45.1%) males	265 (54.0%) females, 225 (45.9%) males	90 (54.8%) females, 74 (45.1%) males	χ^2^ = 2.792 *p* = .425^a^

^a^ Chi‐squared test.

The blood GLU levels of all participants were tested by a glucose meter (Roche, Switzerland) in the morning between 8 a.m. and 9 a.m. after an overnight fast for 10 hr. Then, blood samples (5 ml) were collected in EDTA‐coated tubes while the participants were still in the fasting state, and processed as quickly as feasible (within approximately 3 hr). Plasma was prepared by centrifuging samples for 10 min at 2200 g. The supernatant was aliquoted and stored at −80°C. Samples were only thawed immediately prior to analysis.

The plasma biochemical indicators including TG, TC, ApoA1, ApoB, and HCY were detected by an automatic biochemical analyzer (ICUBIO iMagic‐M7, Shenzhen, China) with the corresponding kits. The potential dementia biomarkers including TREM2, TIMP1, and VLDLR were detected by microplate reader (BioTek‐800TS, USA) using commercially available enzyme‐linked immunosorbent assay (ELISA) kits (Cloud Clone: SEG628Hu and SEA552Hu; Senbeijia biological technology: SBJ‐H1100) following the manufacturer's instructions.

The NFL levels in the plasma were measured by LabEx (Univ‐bio, Shanghai, China) using a Meso Scale Discovery (MSD) electrochemiluminescence method with the corresponding kit (F217X, MSD). Plasma concentrations of Aβ1‐40, Aβ1‐42, and T‐tau were determined by G‐BIO (G‐BIO Biotech, Hangzhou, China) using a single molecule array (Simoa) method with the corresponding Neurology 3‐Plex A kit (N3PA, Quanterix). The APOE genotype was determined by sequencing (WeGene, Shenzhen, China) for SNPs rs7412 and rs429358.

The gender and APOE genotype of test subjects after exclusion (*n* = 77) were compared among different age groups using chi‐squared test. For other demographics values and blood parameters, we performed ANCOVAs with sex and the presence of an APOE ε4 allele included as covariates according to former studies, as APOE and sex have been reported to have effects on some of the dementia biomarkers (Sampedro, Vilaplana, Leon, Alcolea, & Fortea, 2015; Startin, Ashton, Hamburg, Hithersay, & Strdom, 2019). Blood parameters were log‐transformed prior to ANCOVAs. The *η* ^2^ values resulted in ANCOVAs determined the overall effect size of different age groups. If sex and the presence of an APOE ε4 allele showed no confounding effects to certain parameter, then one‐way ANOVA with Bonferroni post‐hoc pairwise comparisons were applied. Otherwise post‐hoc test in each ANCOVA was applied. *p <.05, **p <.01, and ***p <.001 were considered as statistically significant. For correlation analyses between age and each blood parameter, Spearman's rank correlational analysis (Sedgwick, 2014) was used if one of the parameters was not normally distributed, otherwise Pearson's correlation analysis was used. Aβ and T‐tau are proteins that aggregate in the patient's brain with neurodegenerative disease. The ratio of Aβ42 to Aβ40 (Aβ40/42) and the ratio of Aβ42 to T‐tau (Aβ42/T‐tau) in cerebrospinal fluid have been widely accepted as biomarkers of depositions in the brain and early‐stage dementia (Koyama et al., 2012; Park et al., 2019). Thus, they were included in the correlation analyses as well. The heatmaps of correlation analyses were generated using the TBtools software (Chen et al., 2020).

#### 2.2.2 Data preprocessing

Image preprocessing was carried out using a computational anatomy toolbox 12 (CAT12) (http://dbm.neuro.uni-jena.de/cat12/). The voxel‐based morphometry (VBM) Method Flow in CAT12 was shown in Figure S3. We chose initial voxel‐based processing and refined voxel‐based processing to obtain the gray matter maps. T1‐weighted images were all firstly inhomogeneity corrected. The skull and other nonbrain elements were then removed. The images were registered into the standard MNI space using the deformable registration algorithm DARTEL57. An MNI‐registered image, and the images of gray matter were generated with voxel size 1.5 mm^3^ and matrix of 121 × 145 × 121. The gray matter image of each subject was used to construct the deep learning model.

*Z*‐score normalization was performed on the blood parameters.

### 2.3 Deep transfer learning model

First, we used the public dataset to train the convolutional neural network (CNN) model as a pretrained network model. It was used as a starting point to fine‐tune the network with our recruited dataset. Second, we froze the model weights of the convolutional layers so that only the fully connected layers were trainable. This strategy allowed us to train a stable model with less time and data. Then, the MRI features were extracted for subsequent bilinear fusion.

**FIGURE 1:**
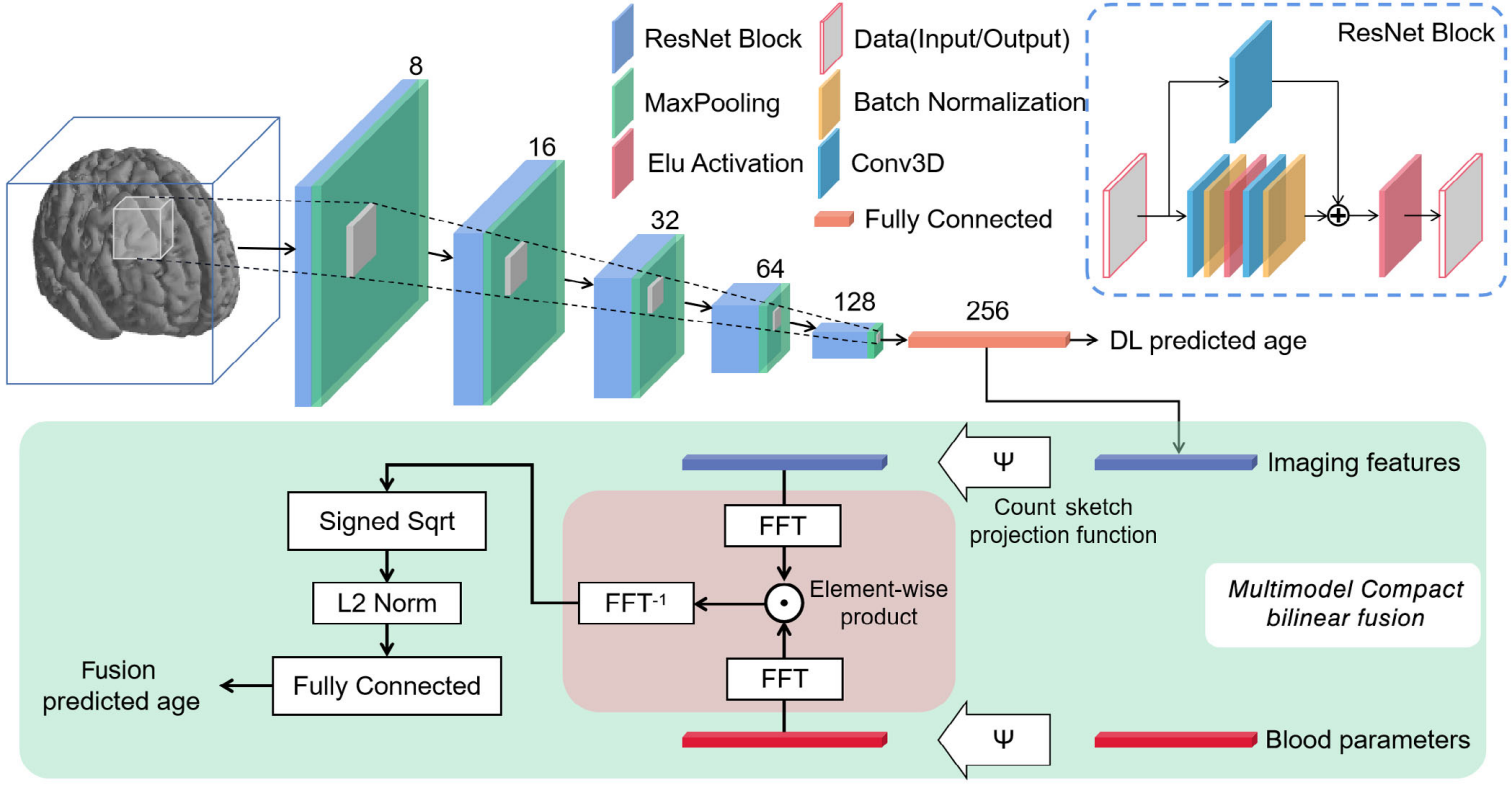
Schematic network architecture and workflow of bilinear fusion. Two hundred fifty‐six‐dimensional features were extracted from brain images through a ResNet network. All MaxPooling layers were designed as stride 2 × 2 × 2 and kernel size 3 × 3 × 3. Imaging features and blood parameters were fused in the Multimodal Compact Bilinear Fusion module. The entire process can be completed by an end‐to‐end cascade network

Our deep‐learning‐based brain age prediction approach was motivated by a pioneering work (Jonsson et al., 2019), which demonstrated that a CNN model trained on MRI scans of healthy elders can achieve high predictive accuracy. A full description of the applied CNN model was presented in Figure 1. It was implemented using Keras with TensorFlow as backend and consisted of five residual blocks, each followed by a max pooling layer of stride 2 × 2 × 2 and kernel size 3 × 3 × 3. The convolutional part of the CNN reduced the input image from size 121 × 145 × 121–128 feature maps of size 4 × 5 × 4. Detailed graphical presentation of the network architecture can be found in Figure S1. We flattened the output from the last convolutional layer and fed it into a fully connected layer which reduced these feature maps down to a feature vector of 256 dimensions. The predicted age was obtained by using the last fully connected layer, which mapped the feature vector to a single output value. The algorithm was optimized by using Adam algorithm with mean absolute error (MAE) loss function and with following parameters: learning rate = 0.0001, decay = 10^−6^, β1 = 0.9, β2 = 0.999, and batch size = 4.

The public dataset was randomly divided into the training (60%), validation (20%), and test (20%) sets. The pretrained network model was built on the training split and the best model was selected based on its performance on the validation set. After the pretrained weights were loaded by deep transfer learning, five‐fold cross validation was conducted to evaluate the prediction performance of CNN model based on our recruited dataset.

The prediction performance of each model was evaluated using MAE and correlation analysis between the predicted brain age and the chronological age. The rules of correlation analysis were the same as mentioned above in Section 2.2.

### 2.4 Bilinear fusion model

#### 2.4.1 Bilinear fusion model

To achieve effective fusion of image features and blood parameters, we found inspiration in the Visual Question Answering tasks (Cadene, Ben‐Younes, Cord, & Thome, 2019). Multimodal bilinear fusion strategies have been recently proposed (Fukui et al., 2016; Yu, Yu, Xiang, Fan, & Tao, 2018) to concatenate the visual and textual representations. Multimodal compact bilinear (MCB) pooling, as a key component in bilinear fusion method, is to obtain a joint representation and calculates the outer product between two vectors. It allows for multiplicative interactions between all elements of both vectors. An MCB model is implemented by projecting the multimodal features to a higher dimensional space and then convolving both vectors efficiently by using an element‐wise product in Fast Fourier Transform space. In our fusion prediction model, the image features (256 features per subject) and the blood parameters (14 features per subject) were used as inputs while the predicted brain age was derived from a fully connected layer at the end of the bilinear fusion model.

#### 2.4.2 Performance comparison with the deep transfer learning model without fusion

The support vector regression (SVR; Drucker, Burges, Kaufman, Smola, & Vapnik, 1996), as a typical machine learning method, was used to build the brain age prediction model based exclusively on blood parameters. SVR aims to construct a linear spacer band in high‐dimensional space based on the training sample set. It counts the distance from the out‐of‐spacer sample to the spacer band into the loss function, and optimizes the model by minimizing the width of the spacer band and the total loss. The radial basis function kernel was used in this study, which transformed the low‐dimensional linear inseparable original features into higher dimensional spaces, making them linearly separable. This enabled the model to fit nonlinear relationships between multiple blood parameters and age. For the hyperparameters *ε* and *C*, we adopted the parameter optimization to discover the optimal hyperparameter automatically. To avoid different models used for the prediction could be a potential confounding effect when comparing the performance between using single modal features or using fusion. We also applied another three different regressors, namely, linear regression, random forest regressor, and Lasso regressor, to compare the regression performance. The linear regression was taken from the fully connected layer without an activation function in the CNN. We utilized univariate linear regression tests for feature selection. The cross‐validation strategy was the same as that described above for CNN model.

Permutation test was used to investigate whether the performance of the fusion model was statistically significantly improved compared with those before fusion. The test was performed between: (1) the model trained on MRI before fusion versus the fusion model, and (2) the model trained on blood parameters before fusion versus the fusion model. For both models, we calculated the errors between the chronological ages and the predicted ages, and obtained two sets of errors. We first calculated the mean values of the two sets, and obtained the true difference of the two mean values. These two sets of error values were then combined into one set and randomly divided equally into two groups, and the difference of the two mean values was calculated as well. We permutated for 999 times and plotted the distribution of the 1,000 (999 fake difference and the true difference) values to test if the true difference was within top 5% (*p* <.05), which indicated that the improvement of fusion model was statistically significant. We completed this permutation test based on the R‐package (https://www.r-project.org/).

To explore the improvement of predictive accuracy after the fusion with blood parameters among different age groups, we evaluated MAE and reduction of MAE. Reduction of MAE was calculated by the formula below:

$$\text{Reduction of}\;\mathrm{MAE}\left(\%\right)=\frac{{\mathrm{MAE}}_{\text{before}}-{\mathrm{MAE}}_{\text{after}}}{{\mathrm{MAE}}_{\text{before}}}\times 100\%$$

Variances of predictive error were calculated separately for different age groups to measure the degree of dispersion of predictive errors.

### 2.5 Feature interpretability analysis

Brain regions visualization and mediation analysis were used to explore the interpretability of the features extracted from the CNN model. Gradient‐weighted Class Activation Mapping (Grad‐CAM) method (Selvaraju et al., 2017) was used to extract the attention map showing which brain areas contribute significantly to age prediction. The attention maps from all samples were averaged, resulting in an average attention map. Anatomical automatic labeling (AAL) atlas (Tzourio‐Mazoyer et al., 2002) was superimposed on the average attention map and obtained voxel values from the total 90 regions indicated in AAL. We calculated the sum of voxel values as well as the number of voxels in each region, and their ratio (sum of voxel values/number of voxels) was identified as importance score (Wang et al., 2019). We did not consider the difference between the left and right brain, so the average number of importance scores in the same brain region located in different hemibrains was taken as the importance score for that brain region. Forty‐five importance scores were finally obtained. Furthermore, we normalized the minimum to maximum values of importance score to range from 0 to 1. The importance score of the top 8 brain regions accounted for more than 40% of the importance score for all brain regions. Therefore, only the top 8 brain regions with the highest importance score were involved in subsequent analyses. Brain anatomical features can be divided into features of subcortical and cortical regions. There were five subcortical regions and three cortical regions in the top 8 brain regions. The FreeSurfer software (https://surfer.nmr.mgh.harvard.edu/fswiki/) package was used to estimate the volume of subcortical regions. The surface area and gray matter volume (GMV) of cortical regions were estimated by FreeSurfer as well. Eleven features were included in the brain anatomical features of each subject. The correlation analyses between age‐related blood parameters and brain anatomical features were conducted by using the same method as mentioned above in Section 2.2. The features which were not significantly associated with chronological age were excluded in this analysis.

To explore whether there were interactions between brain anatomical features and chronological age or blood parameters and chronological age, mediation analyses (Imai, Keele, & Tingley, 2010) were applied to these variables that were found to have significant linear relationships with age. The first step was to test whether blood parameters mediate the relationship between brain anatomical features and age. We screened variables from brain anatomy features by linear regression model. Age was used as the dependent variable in linear regression model, only those features whose regression coefficients were statistically significant (*p* <.05) were selected (*n* = *N*_screen_). For mediators, that is, blood parameters, no screening was performed (*n* = 13). Next, we explored the mediating effects by using R‐package. A total of 13 × *N*_screen_ analyses were performed. The second step was to test whether brain anatomical features mediate the relationship between blood parameters and age. Compared to the first step, the roles of brain anatomical features (*n* = 11) and blood parameters were interchanged. The screening rules for blood parameters (*n* = *N*′_screen_) and mediation analyses for each variable were the same as above. A total of 11 × *N*′_screen_ analyses were performed. Total, direct, and mediation effects were analyzed by using 1,000 bootstraps with bias‐corrected 95% CI. We used the Benjamini–Hochberg method to correct for multiple comparisons in mediation analyses (Benjamini & Hochberg, 2000).

Further, we performed PCA to explore which blood parameters influence brain age prediction most. PCA is a dimension‐reducing method that creates a new coordinate space according to variance and singular value decomposition algorithm (Ringnér, 2008). PCA can projects high‐dimensional data into low‐dimensional space while maintaining its principal components. PCA steps were performed using sklearn toolkit in Python 3.6 (https://www.python.org/). Specifically, we stitched the matrix of image feature and the blood parameters to obtain a 270‐dimensional feature matrix. *Z*‐score standardization was performed in each feature. The first two principal components with the largest variance were selected to set a coordinate space. Then the 270‐dimensional features were projected into the coordinate space and obtained their new feature values in the first two principal components.

## 3 RESULTS

### 3.1 Demographic information

The characteristics of the recruited subjects were described in Table 1. The recruited dataset consisted of 77 (47 females/30 males) cognitively normal elderly individuals from Chinese population after exclusion. Their ages ranged from 50 to 85 years with a mean value of 62.1 and standard deviation (*SD*) of 8.6 years. The recruited subjects were divided into three groups by age: the 50–60 group, the 60–70 group and the 70–85 group. One‐way ANOVA and post‐hoc analyses were performed to compare the MMSE and MoCA‐B scores in these groups, and no statistically significant difference was found.

Description of the sMRI datasets derived from public databases was given in Table 2. In total, the public dataset consisted of 1,481 (822 females/659 males) cognitively normal elderly individuals. Their ages ranged from 50 to 85 years with a mean age of 68.0 and *SD* of 8.6 years.

### 3.2 Correlations between blood parameters and chronological age

The median values and ranges of the blood parameters in each group of our subjects were listed in Table S1. One‐way ANOVA and post‐hoc analyses were performed to compare the group difference. The levels of GLU were significantly higher for the 70–85 group compared to the 50–60 group or the 60–70 group. The HCY levels showed significant differences between the 50–60 and 60–70 groups, and between the 50–60 and 70–85 groups. The concentrations of NFL showed significant differences in each group. The plasma Aβ40 level was significantly higher in the 60–70 group compared to the 50–60 group.

The results of normality test were listed in Table S1. There were three parameters, Aβ40, Aβ42, and T‐tau that conformed to the normal distribution while others were non‐normal. In addition, chronological ages of recruited subjects were not normally distributed (*p* = .023), so Spearman's rank correlation analyses were performed to determine the correlation between age and blood parameters.

**FIGURE 2:**
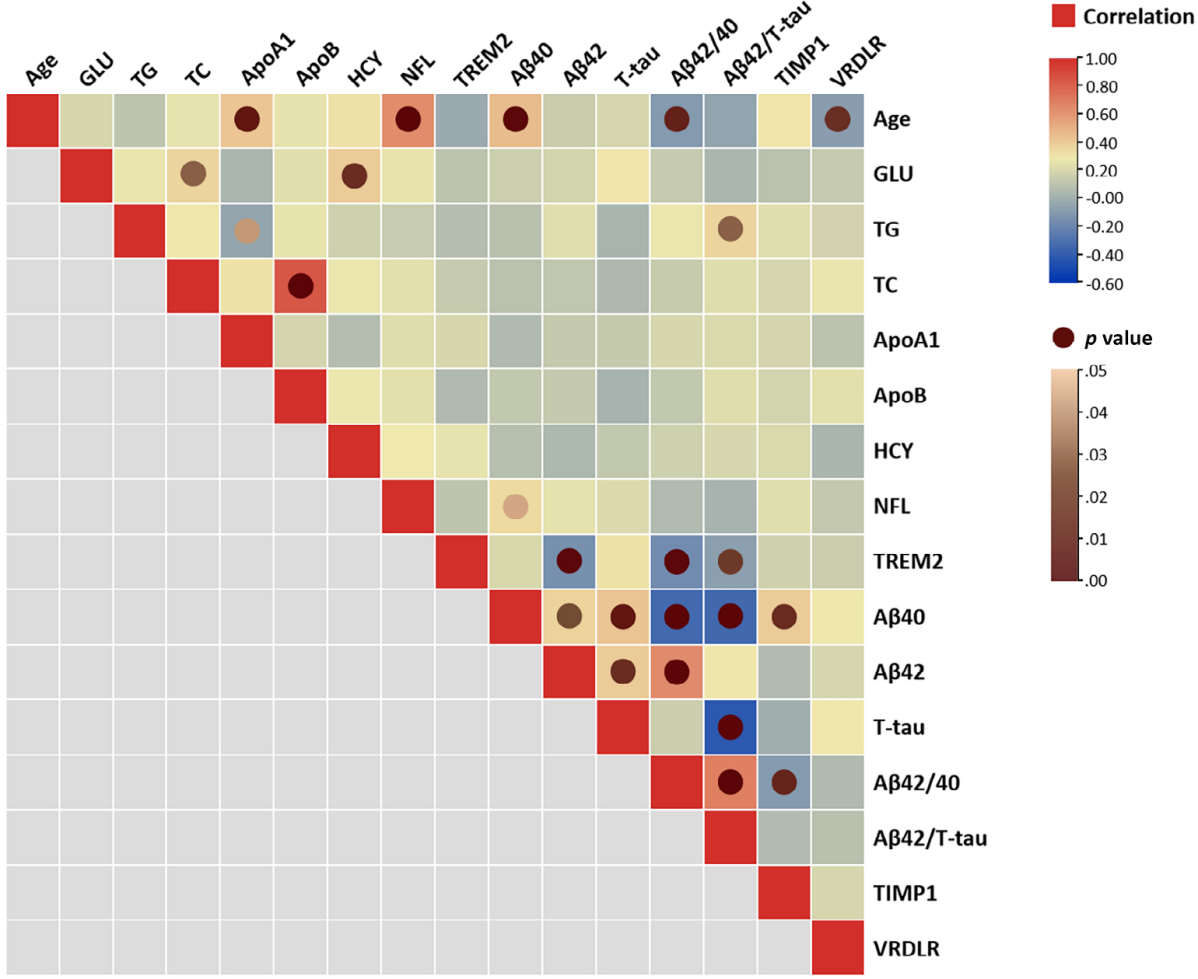
Correlations between concentrations of blood parameters and chronological age. The squares colored with gradient color from red to blue were for Spearman's rank correlation coefficients, and circle nodes with light brown to dark brown were for *p* value with significance. GMV, gray matter volume

The results of correlation analyses were provided in Table S2. As shown in the heatmap (Figure 2), only the correlations with statistical significance (*p* <.05) were labeled with circle nodes. Significant positive correlations were found between: GLU and TC, GLU and HCY, Aβ42/ T‐tau and TG, ApoB and TC, Aβ40 and NFL, Aβ42 and Aβ40, T‐tau and Aβ40, TIMP1 and Aβ40, T‐tau and Aβ42, Aβ42 and Aβ42/40, Aβ42/40 and Aβ42/T‐tau. Significant negative correlations were found between: ApoA1 and TG, Aβ42 and TREM2, Aβ42/40 and TREM2, Aβ42/T‐tau and TREM2, Aβ42/40 and Aβ40, Aβ42/T‐tau and Aβ40, Aβ42/T‐tau and T‐tau, TIMP1 and Aβ42/40.

**FIGURE 3:**
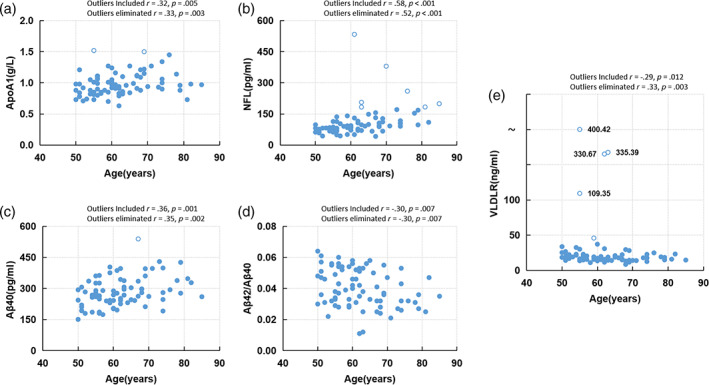
Scatterplots of age‐associated blood parameters over chronological age. The concentrations of (a) ApoA1, (b) NFL, (c) Aβ 40, (d) Aβ 42/40, (e) VLDLR. Outliers are denoted by hollow circles. (*r* = Spearman's rank correlation coefficient, *p* = two‐sided *p* value)

Notably, we identified significant positive correlations between chronological age and blood parameters of ApoA1, NFL, Aβ40, and negative correlations between chronological age and blood parameters of Aβ42/40, VLDLR. The results of the correlation analyses revealed the potential of blood data for age estimation. Scatterplots were shown in Figure 3 to present the distribution of these blood biochemical indicators over chronological age. We performed outlier detection and found that excluding the outliers did not change the significance of the correlation.

### 3.3 Brain age prediction performance of the deep transfer learning model

**FIGURE 4:**
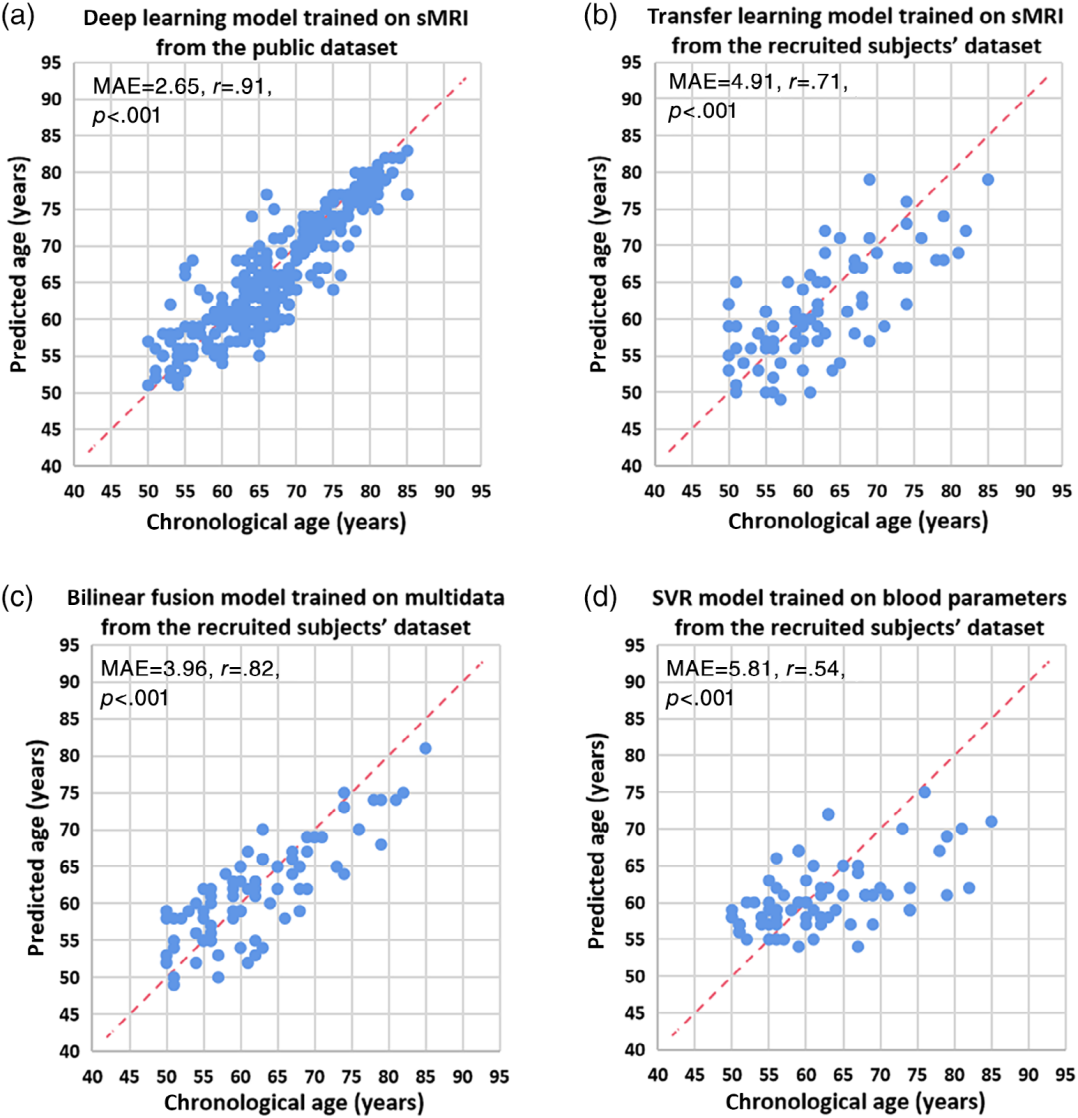
Accuracy of brain age prediction models. Scatterplot of chronological age (*x*‐axis) and predicted age (*y*‐axis) from (a) deep learning model trained on sMRI from the public dataset, (b) transfer learning model trained on sMRI from the recruited subjects' dataset, (c) bilinear fusion model trained on sMRI and blood parameters from the recruited subjects' dataset, (d) SVR model trained on blood parameters from the recruited subjects' dataset. Blue circles represent the subjects, and the dashed red line represents the perfect prediction. The model performance, including MAE (years) and results of correlation analysis were provided on the left corner of each plot

Performance of our trained deep learning model on the test set (*n* = 297) from public dataset showed an MAE value of 2.65 years (Figure 4a). The age distribution of the test set did not conform to a normal distribution (*p* = .004). Spearman's rank correlation analyses were performed and found a good correlation (*r* = .91, *p* <.001) between chronological age and predicted brain age. The pretrained model weights were then loaded into the CNN and fine‐tuned with our recruited subjects. The performance measured on the recruited dataset showed an MAE of 4.91 years (Figure 4b) with a relatively high Spearman's rank correlation (*r* = .67, *p* <.001). For comparison, the deep learning model trained on our recruited dataset without pretrained weights showed an MAE of 6.03 years with a Spearman's rank correlation coefficient of .42 (*p* <.001).

### 3.4 Brain age prediction performance of the fusion model

As shown in Figure 4c, the multimodal bilinear fusion of both brain MRI and blood parameters resulted in a better prediction performance (MAE, 3.96 years; *r* = .76, *p* <.001). In addition, the fusion model showed a significantly lower MAE in predicting brain age than the MAE of model trained on sMRI only after a permutation test (*p* = .048). The SVR model based exclusively on blood parameters achieved a worse performance (MAE, 5.81 years; *r* = .53, *p* <.001) than the fusion model. The permutation test of the SVR model versus the fusion model also indicated a significant improvement in the fusion model (*p* = .002). The scatterplot was shown in Figure 4d and these results can be found in Table 3. Among all, the bilinear fusion model showed the highest prediction performance on our recruited dataset. The results of different models used for the prediction using single modal features or using fusion were listed in Table 4. The experimental results showed that predictive accuracy of fusion model with different regression methods was generally better than that of the model trained on single modal features. It can be concluded that the improvement of predictive accuracy was due to multimodal features rather than different regressors.

**TABLE 3:** Age predictive accuracy

Dataset	Features	Method	MAE (years)	Spearman's rank correlational analysis
Public data (*N* = 1,481)	MRI only	Deep learning	2.65	*r* = .91 *p* <.001
Recruited subjects' data (*N* = 77)	MRI only	Deep learning	6.03	*r* = .42 *p* <.001
Recruited subjects' data (*N* = 77)	MRI only	Transfer learning and deep learning	4.91	*r* = .67 *p* <.001
Recruited subjects' data (*N* = 77)	Blood parameters only	Support vector regression	5.81	*r* = .53 *p* <.001
Recruited subjects' data (*N* = 77)	Bilinear fusion of MRI and blood parameters	Bilinear fusion with linear regression	3.96	*r* = .76 *p* <.001

**TABLE 4:** Prediction performance of different regressors on different modal features

Features	Feature extraction	Features shape	Regressors	MAE (years)	Spearman's rank correlational analysis
MRI only	ResNet	256 × 1	Linear	4.91	.67
	ResNet	256 × 1	Support vector	5.49	.61
	ResNet	256 × 1	Random forest	4.88	.69
	ResNet	256 × 1	Lasso	5.12	.65
Blood parameters only	None	14 × 1	Linear	5.90	.53
	None	14 × 1	Support vector	5.81	.54
	None	14 × 1	Random forest	5.60	.57
	None	14 × 1	Lasso	6.10	.50
Bilinear fusion of MRI and blood parameters	ResNet + MCB	14 × 1	Linear	**3.96**	**.76**
	ResNet + MCB	14 × 1	Support vector	4.60	.74
	ResNet + MCB	14 × 1	Random forest	4.76	.72
	ResNet + MCB	14 × 1	Lasso	4.64	.71

*Note:* Bold values signifies that *p‐*value is <.001

**FIGURE 5:**
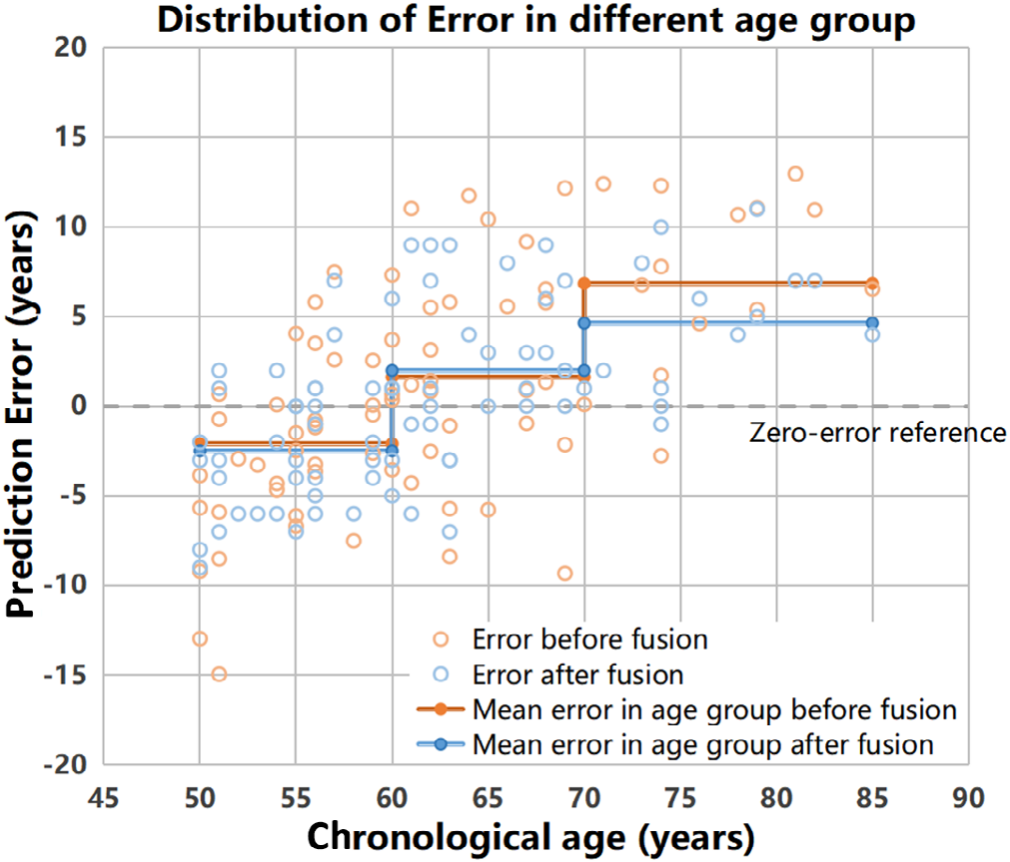
Distribution of age prediction error in different age groups. Each step colored in orange (before fusion) or blue (after fusion) line indicated the mean error in that age group. The gray dashed line indicates the zero‐error reference

As listed in Table 5, improved performance was shown in all three age groups. Among these three groups, the 70–85 age group showed the best improvement after incorporating the blood parameters. As shown in Figure 5, the mean prediction error of the fusion model, compared with the model before fusion, was significantly lower in the 70–85 age group.

**TABLE 5:** Predictive accuracy in different age groups

Age group	Features	Variance of predictive error (years)	Mean absolute error (MAE, years)	Reduction of MAE (%)
50–60 (*N* = 33)	MRI only	11.40	4.03	—
	Bilinear fusion of MRI and blood parameters	6.30	3.63	9.77
60–70 (*N* = 30)	MRI only	14.00	4.83	—
	Bilinear fusion of MRI and blood parameters	9.78	3.93	18.62
70–85 (*N* = 14)	MRI only	16.59	7.14	—
	Bilinear fusion of MRI and blood parameters	12.48	4.78	33.00

### 3.5 Feature interpretability analyses

#### 3.5.1 Visualization of important brain regions in age prediction

**FIGURE 6:**
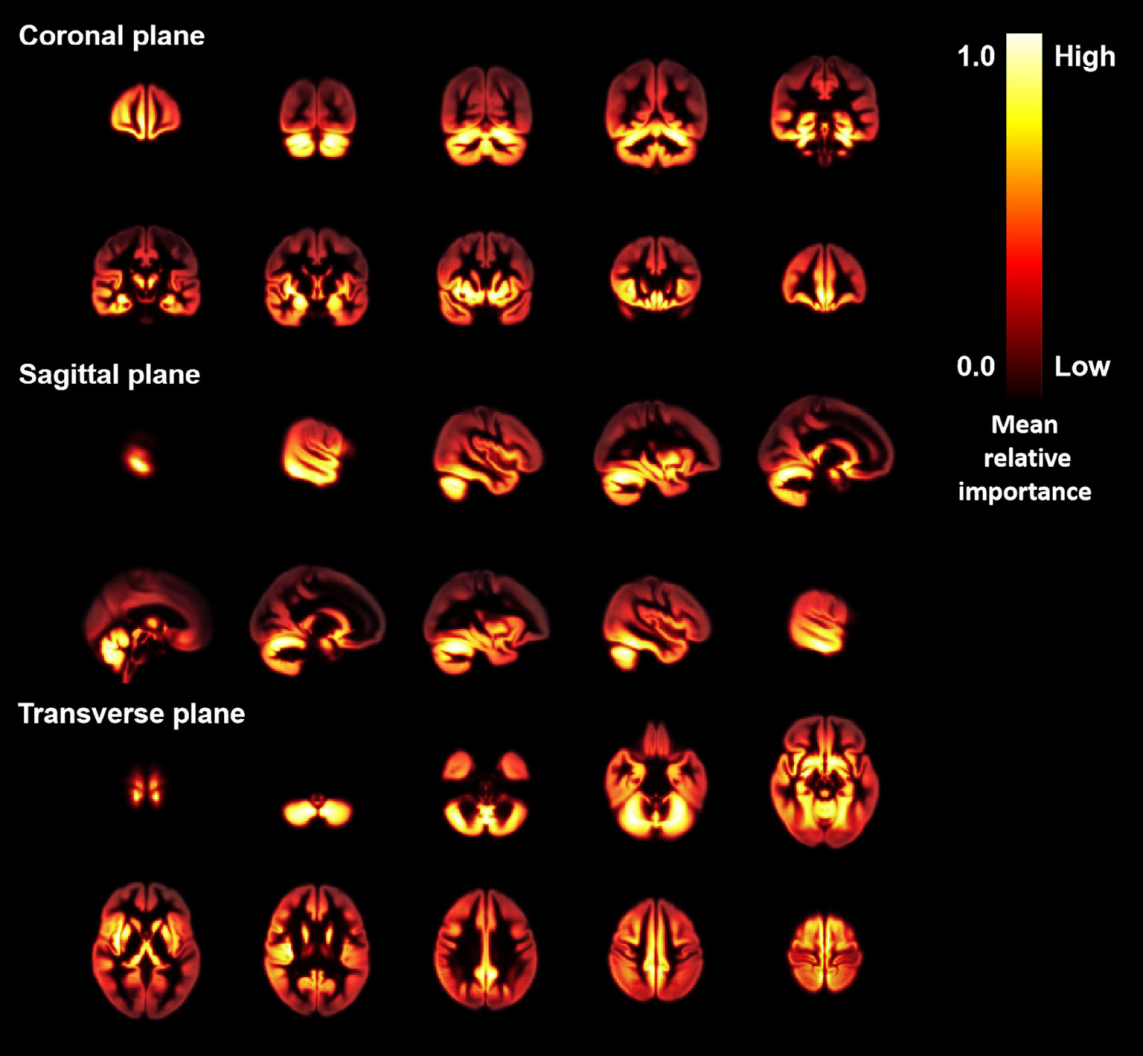
Effective brain regions for contributing the age prediction. Grad‐CAM attention map overlaid on a brain template from coronal plane, sagittal plane, and transverse plane. Areas highlighted with gradient color from yellow to red showed the effective brain regions for age prediction

As shown in Figure 6, the attention map highlighted the areas that were effective for age estimation, which mainly included brain structures that belong to the limbic system and basal ganglia. The top 8 anatomical regions that contributed to the age prediction were listed in Table 6. Amygdala was the region with the highest contribution. The 45 anatomical regions in AAL ranked by the importance score were listed in Table S3.

**TABLE 6:** Top 8 anatomical brain regions ranked by the importance in the age prediction model

Brain region	Size (voxels)	Importance (normalized)
Amygdala	948	1.00
Pallidum	1,291	0.99
Olfactory	1,245	0.93
Putamen	3,984	0.92
Hippocampus	4,081	0.89
Thalamus	4,374	0.87
Fusiform	11,463	0.85
Parahippocampus	5,387	0.85

**FIGURE 7:**
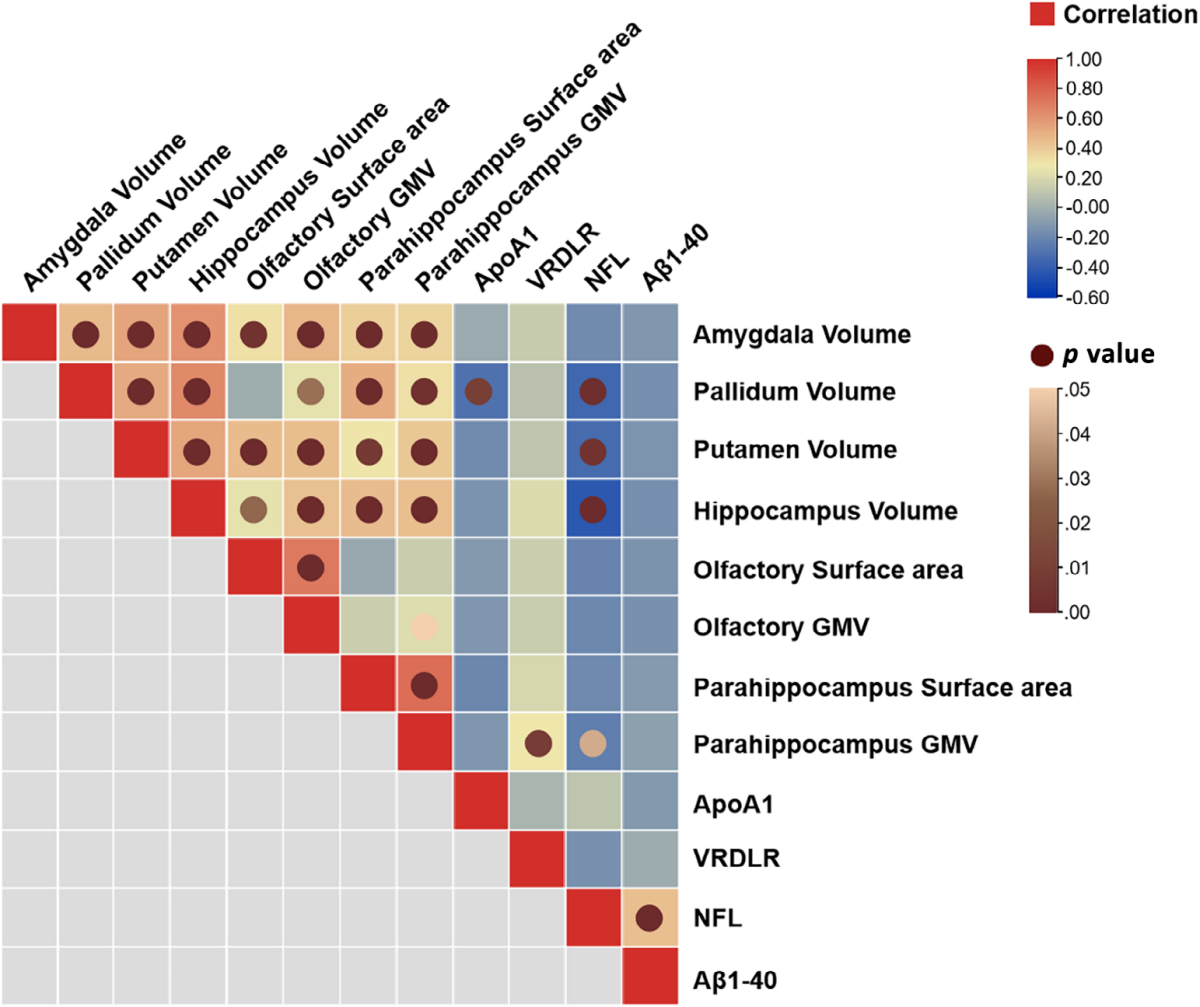
Correlation between age‐related brain region features and blood parameters. The squares colored with gradient color from red to blue were for correlation coefficients, and circle nodes with light brown to dark brown were for *p* value with significance. GMV, gray matter volume

As shown in the heatmap (Figure 7), a significant positive correlation was found between VLDLR and parahippocampus GMV, while significant negative correlations were found between ApoA1 and pallidum volume, NFL and pallidum volume, NFL and putamen volume, NFL and hippocampus volume, NFL and parahippocampus GMV. The results of the correlation analyses were provided in Table S4. Furthermore, the correlations between different brain anatomical features revealed a complex interplay between the brain regions.

#### 3.5.2 Mediation analysis between brain MRI features and blood parameters

**FIGURE 8:**
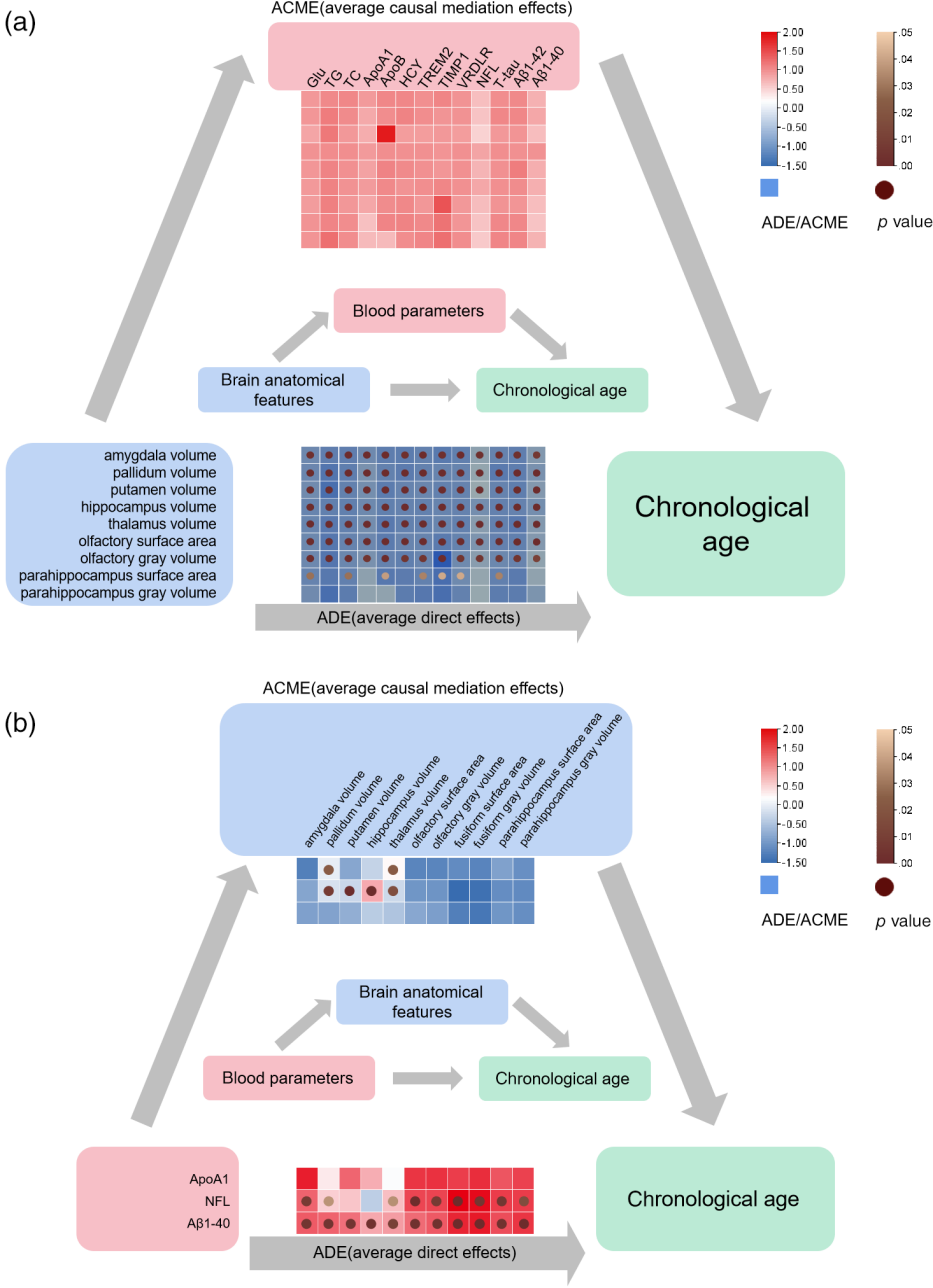
Mediation analysis. (a) The ACE and ACME results of brain anatomical features on chronological age via blood parameters. (b) The ADE and ACME results of blood parameters on age via brain anatomical features. ACME, average causal mediation effects; ADE, average direct effects. The squares colored with gradient color from red to blue were for ADE or ACME values, and circle nodes with light brown to dark brown were for *p* value with significance

As shown in Figure 8a, in most cases, the brain anatomical features showed direct effects on age. Meanwhile, four partial mediation effects were identified. To be specific, TIMP1 partially mediated between olfactory GMV and chronological age. NFL partially mediated between putamen volume, thalamus volume and chronological age. Aβ40 partially mediated between thalamus volume and chronological age. Figure 8b showed that the ApoA1, NFL, and Aβ40 directly affected age in most cases. Besides, pallidum volume and thalamus volume partially mediated between ApoA1 and age. Pallidum volume, putamen volume, hippocampus volume, and thalamus volume partially mediated between NFL and age.

#### 3.5.3 Main factors of blood parameters for brain age determined by PCA

**FIGURE 9:**
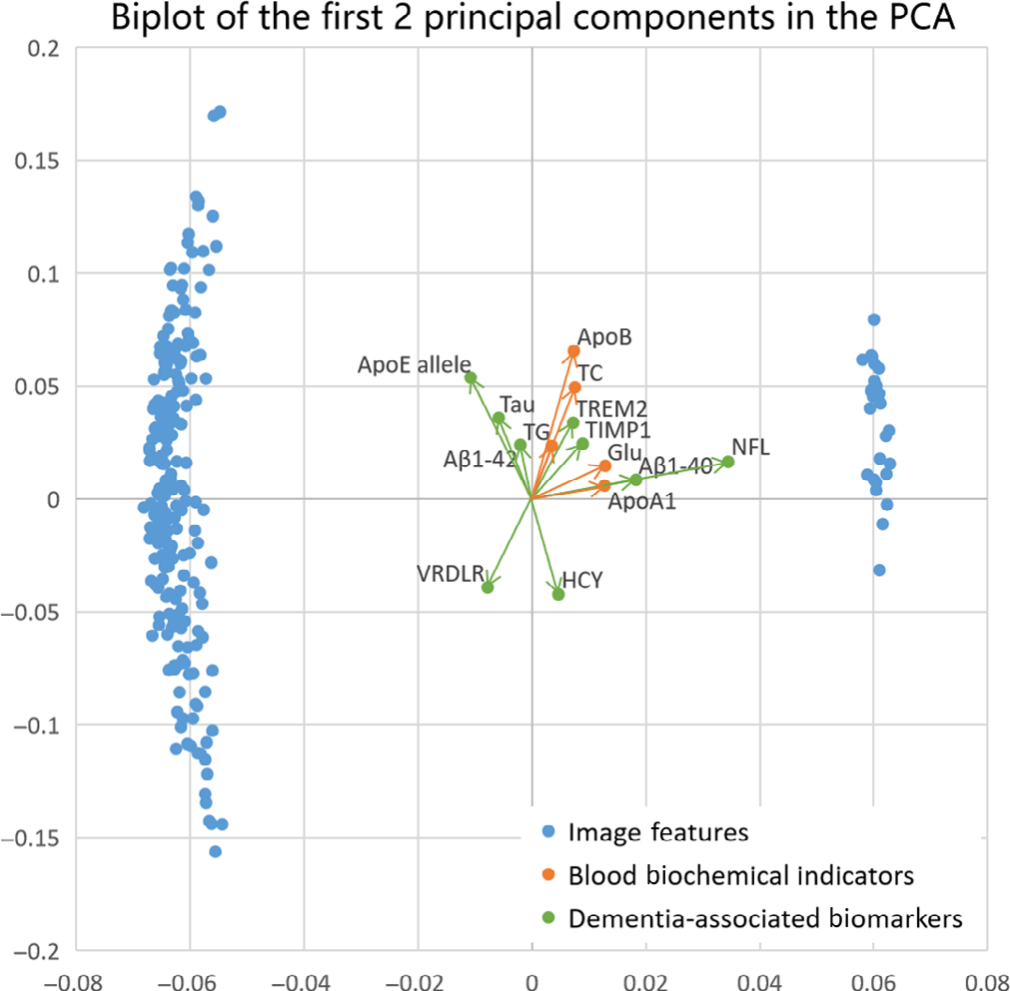
Principal component analysis (PCA). Biplot of the first two principal components in the PCA, accounting for the greatest variance, with blood parameters (including five blood biochemical indicators and nine dementia‐associated biomarkers) labeled. Arrows showed the contribution of original variables to the principal components

The results of PCA revealed that image features scores on principal components were higher than blood parameters, but the direction of features on principal component was different between image features and blood parameters. According to Figure 9, ApoE allele, lipid parameters (including ApoB and TC) and VRDLR had high scores on the second principal component. They showed different sizes and different directions vectors in the coordinate space formed by the first two principal components. This further illustrated that their potential information was different. In contrast, NFL, which had the highest correlation with image features, had similar components to image features groups on principal direction.

## 4 DISCUSSION

To the best of our knowledge, this is the first study to demonstrate that integration of blood parameters and brain sMRI data yields higher brain age predictive accuracy in elderly population. Subsequent analyses confirmed the improvement by bilinear fusion for different age groups. Feature interpretability analyses showed important brain regions that contribute to the prediction. Direct and indirect effects between blood parameters, anatomical features and age had been discovered.

### 4.1 Deep transfer learning improves brain age prediction

One possible limitation in constructing the brain age prediction model is the small sample size. Therefore, we applied the deep transfer learning method in our study. The model was pretrained with available data from public dataset, and then it was applied to our recruited dataset. The deep transfer learning obviously improved the model performance on our recruited dataset. Our pretraining model using the same method achieved higher accuracy than the published model (ages 19–75) (Jonsson et al., 2019), which might be due to a more specific age range (ages 50–85) that we were targeting. A hypothesis of transfer learning is that a CNN which already proficient at one site only needs a small adjustment to adapt data from a new site. The CNN trained on larger datasets can extract rich and effective brain structural features that may be common among different sites. The heterogeneity inherent in different sites can be adapted with small adjustments, so that the CNN can train on small datasets more quickly and stably. Still, further confirmation is required on larger datasets with a wider age range for the multimodal fusion model based on brain MRI and blood parameters.

A kind of explanation about CNN model validity investigated in our work was to highlight the brain regions that contribute to age prediction. In accordance with a former report based on MRI data only, the brain regions, including amygdala, hippocampus and thalamus were relatively effective for age prediction (Wang et al., 2019) and their morphometric changes were often detected during aging process (Oschwald et al., 2019). These three brain regions are key components of the brain limbic system, which are responsible for behavioral and emotional responses, and consolidating memories. Relationships between cognitive impairment and atrophy of these brain regions have been reported, even before diagnoses (Štěpán‐Buksakowska et al., 2014; Wachinger, Salat, Weiner, & Reuter, 2016). This might be an explanation why the age gaps between the predicted brain age and the chronological age were reported to be greater in cases with higher dementia risk (Cole & Franke, 2017). Also, pallidum and putamen that belong to the brain basal ganglia were identified in our report, suggesting a possible application of our model in screening neuronal disorders with mobility dysfunction such as Huntington and Parkinson's disease.

### 4.2 Fusion with blood parameters further improves brain age prediction

Since blood biochemical indicators and dementia‐associated biomarkers are also linked to one's brain aging and health state, data from blood parameters also contribute to the brain age prediction. Indeed, the SVR model generated using blood parameters obtained a reliable performance. Furthermore, the model fused with blood and sMRI data achieved higher predictive accuracy than model based solely on sMRI, and performed as well as the model from former reports (Franke & Gaser, 2019; Jonsson et al., 2019).

It was noteworthy that the improvement was more effective in elder age group. One of the most likely reasons is that the blood biochemical indicators and dementia‐associated biomarkers we selected were to evaluate the whole‐body and brain health status of the recruited subjects. Five biochemical indicators including GLU, TG, TC, ApoA1, and ApoB were measured to evaluate the glucose and lipid metabolism of the subjects. Other seven blood parameters are potential risk factors or biomarkers of cognitive impairment (Fitz et al., 2015; Li & Mielke, 2019; Liu et al., 2018; Mattsson, Cullen, Andreasson, Zetterberg, & Blennow, 2019; Smith & Refsum, 2016; Yao et al., 2018). These blood parameters were more closely associated with brain aging at older ages.

The linear relationship between brain age and chronological age in healthy people have been observed in many studies (Bashyam et al., 2020; Cole, 2020; Feng et al., 2020). However, some studies also reported a nonlinear relationship between brain age and chronological age (Niu et al., 2020), in which the brain age tended to be underestimated for older subjects and overestimated for younger ones. Such systematic bias may arise from regression toward the mean (Le et al., 2018; Liang, Zhang, & Niu, 2019) or from the non‐Gaussian distribution of subjects' age (Smith, Vidaurre, Alfaro‐Almagro, Nichols, & Miller, 2019). Similarly, the predicted brain age of 70–85 age group in our study was underestimated. Although our proposed fusion model successfully reduced the underestimation in the 70–85 age group, further studies are required to better correct for the systematic bias in regression model.

### 4.3 Mediation analysis and PCA reveal that the multimodal information are independent and nonoverlapping

The cross‐information benefits from multiple types of data help reveal important links that cannot be detected by single‐modality data. Since the bilinear fusion model worked better than the sMRI model, blood parameters and sMRI might be complementary to each other. In the subsequent mediation analysis, we studied whether blood parameters act as mediators that transmit the effect of brain features on chronological age, and whether brain anatomical features mediate the relationship between blood parameters and age. Some partial mediation effects of the brain anatomical features and blood parameters on age were identified. However, in most cases, they showed significant direct effects on age instead of mediating effects. This indicated that most information from blood parameters and brain sMRI does not have causal pathways, either from blood parameters to brain sMRI features to brain age, or from brain sMRI features to blood parameters to brain age. Some blood parameters including GLU, TC, and TG are sensitive indicators expressing various health states. For example, inflammation and metabolic abnormalities have been successfully used in age prediction tasks (Putin et al., 2016). For dementia biomarkers such as Aβ and T‐tau, previous studies demonstrated that in the cerebrospinal fluid or blood they change far before the onset of neurodegenerative symptoms, which may have not been detectable by imaging method yet (Bateman, Xiong, Benzinger, Fagan, & Goate, 2012). On the other hand, it is well‐known that the brain structural information obtained by brain images is rich and effective. Accelerated atrophy in brain regions implies accelerated aging. This is important information that cannot be captured from blood. Blood parameters and brain sMRI features showed independent information so that they can directly contribute to the performance of predicting brain age respectively.

A similar conclusion can be drawn from PCA. The brain sMRI features and most blood parameters showed components of different sizes and orientations, revealing that their roles do not overlap. We found that apolipoprotein E genotype and lipid parameters play important roles in the prediction of brain age from PCA results. This is reasonable because ApoB variants have been found to be directly related to AD risk (Wingo et al., 2019), also to the degree of brain aging. Meanwhile, higher levels of TC have been reported to be associated with decreased cognitive performance in normal elderly adults (Stough, Pipingas, Camfield, Nolidin, & Scholey, 2019). What is more, there was a mutual influence between apolipoproteins and TC. It has been found that aging‐related processes can substantially impact the role of lipid‐related genes (including ApoB and ApoE allele) in regulation of TC and onset of cardiovascular disease (Kulminski et al., 2013). This indicates that apolipoproteins and TC can reflect the brain aging through the blood circulation system.

We noticed that NFL and ApoA1 have a strong correlation with some brain anatomical features while there were causal mediation effects between them. This means that they provide duplicated information in the brain age prediction, but it is indeed common in many studies. As a potential biomarker for neuronal axonal damage, the plasma NFL was particularly prominent among all blood parameters in our study. A strong positive correlation of NFL levels with age was found in our recruited subjects, which was consistent with results from two other studies on healthy participates in a similar age range (ages >50; Khalil et al., 2020; Wagen et al., 2020). They also demonstrated that NFL levels were inversely correlated with whole brain volume and positively correlated with brain atrophy. In our study, the correlations between NFL and typical brain regions were analyzed specifically, and NFL was found to have significant negative correlations with the volume of pallidum, putamen, hippocampus, and parahippocampus. From results of PCA, the proportion of NFL and Aβ40 in principal components were similar to the proportion of image features. There are studies reporting correlations between brain morphological changes and some of the biomarkers that we used. For instance, NFL levels were inversely correlated with whole brain volume (as we mentioned in the discussion); Aβ positivity was associated with smaller gray matter volumes (Mattsson et al., 2015). Thus, NFL may provide repeated information during fusion prediction, but this demonstrated the importance of NFL as an indicator of brain health status assessment.

In this study, the information provided by blood parameters and brain structural features were independent and nonoverlapping in prediction tasks, which might be an explanation why the fusion of brain sMRI and blood parameters enhanced the predictive accuracy of our model.

Our work provides a clinically adaptable strategy for incorporating routinely available data from blood biochemical and MRI to assess accurate brain age. Besides, it has significant transformational potential beyond brain age prediction. The effective brain regions and the blood parameters highlighted by our model suggest the prospect of early screening across a spectrum of neurodegenerative diseases, such as Huntington, Parkinson, and AD. It may be of interest in future studies to inspect whether the high‐contributing features from the currently presented framework may change follow the neurodegenerative disease progress. In such cases, our model can aid in the noninvasive monitoring of disease development.

### 4.4 Limitations

There may be some possible limitations in this study. First, the sample size of recruited data was small. We thus applied deep transfer learning to lower the concern. Indeed, our model based on deep transfer learning showed improved performance on brain age prediction. For subsequent research and practical clinical application, future longitudinal studies with a larger sample size are warranted to confirm these findings. Correlation and mediation analyses helped to analyze the relationship between different parameters and chronological age, but the mechanism remains unclear.

## 5 CONCLUSION

For the first time, we presented a brain age prediction model with improved performance by deep transfer learning and multimodal fusion of the data from brain sMRI and blood parameters in the Chinese elderly. Compared with other models based solely on brain MRI or blood parameters, the bilinear fusion model achieved the highest accuracy in age prediction. The prediction performance of elderly age group was significantly improved after the fusion of blood parameters. The subsequent mediation analysis discovered direct effects of blood parameters and anatomical features on age in most cases, supporting our hypothesis that brain MRI and blood parameters provide nonoverlapping information which contributed to the performance of fusion model. Our findings show promising potential to be applied in evaluating brain health status for Chinese populations.

## CONFLICT OF INTEREST

The authors declare there are no conflict of interest.

## Supporting information


**Figure S1** Detailed graphical representation of the network architecture.

**Figure S2** The public dataset selection strategies and dataset's age distribution. (a) The collected criteria for ADNI, IXI, and OASIS‐3 database, (b) age distribution in public dataset, (c) age distribution in our recruited dataset. HC, health control.

**Figure S3** CAT processing pipeline.

**Table S1** Concentrations of blood biochemical indicators and dementia biomarkers for each group and group comparisons.
**Table S2.** Relationships between age and blood indicator concentrations.
**Table S3.** The 45 anatomical brain regions ranked by the importance in the age prediction model.
**Table S4.** Correlations between brain anatomical features and age‐related blood parameters.

## Data Availability

The code for brain age prediction is available from the authors upon request. The datasets generated by three public databases used in this study are available via the Alzheimer's Disease Neuroimaging Initiative (http://adni.loni.usc.edu/data-samples/access-data/), the Information eXtraction from Images (http://brain-development.org/ixi-dataset/) and the Open Accessible Summaries In Language Studies (https://oasis-database.org/). The recruited participants’ datasets generated and analyzed in the present study will be made available from the corresponding author to other scientists on request in anonymized format and according to data protection policy in the ethics agreement.
