# Supplementary figures and images for: Deep transfer learning of structural magnetic resonance imaging fused with blood parameters improves brain age prediction
Source: Hum Brain Mapp. 2021 Dec 16;43(5):1640–56. doi: 10.1002/hbm.25748 (PMC8886664; doi:10.1002/hbm.25748)

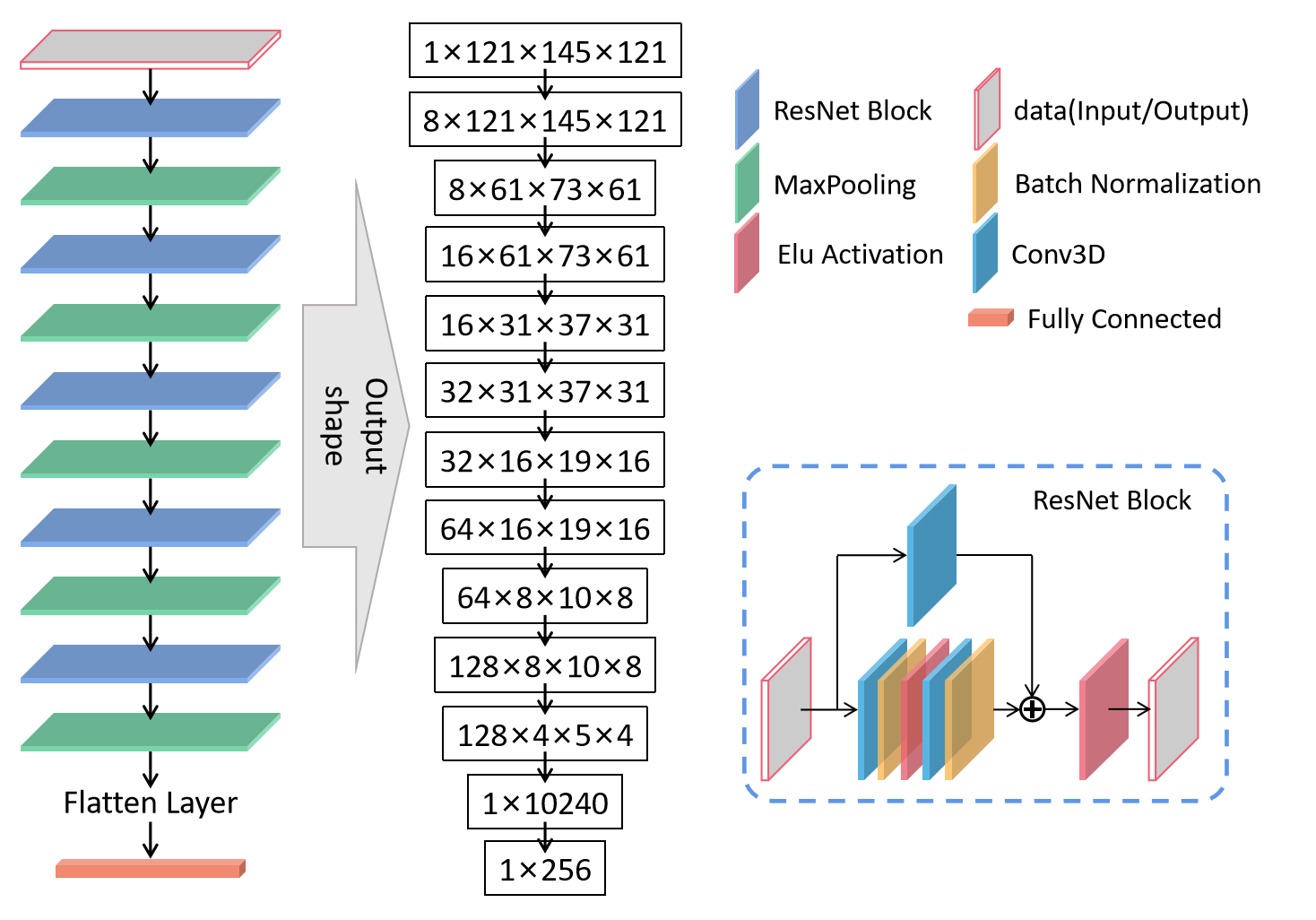

Supplement: Supplementary file 1 — Figure S1 Detailed graphical representation of the network architecture. [file HBM-43-1640-s002.tif]

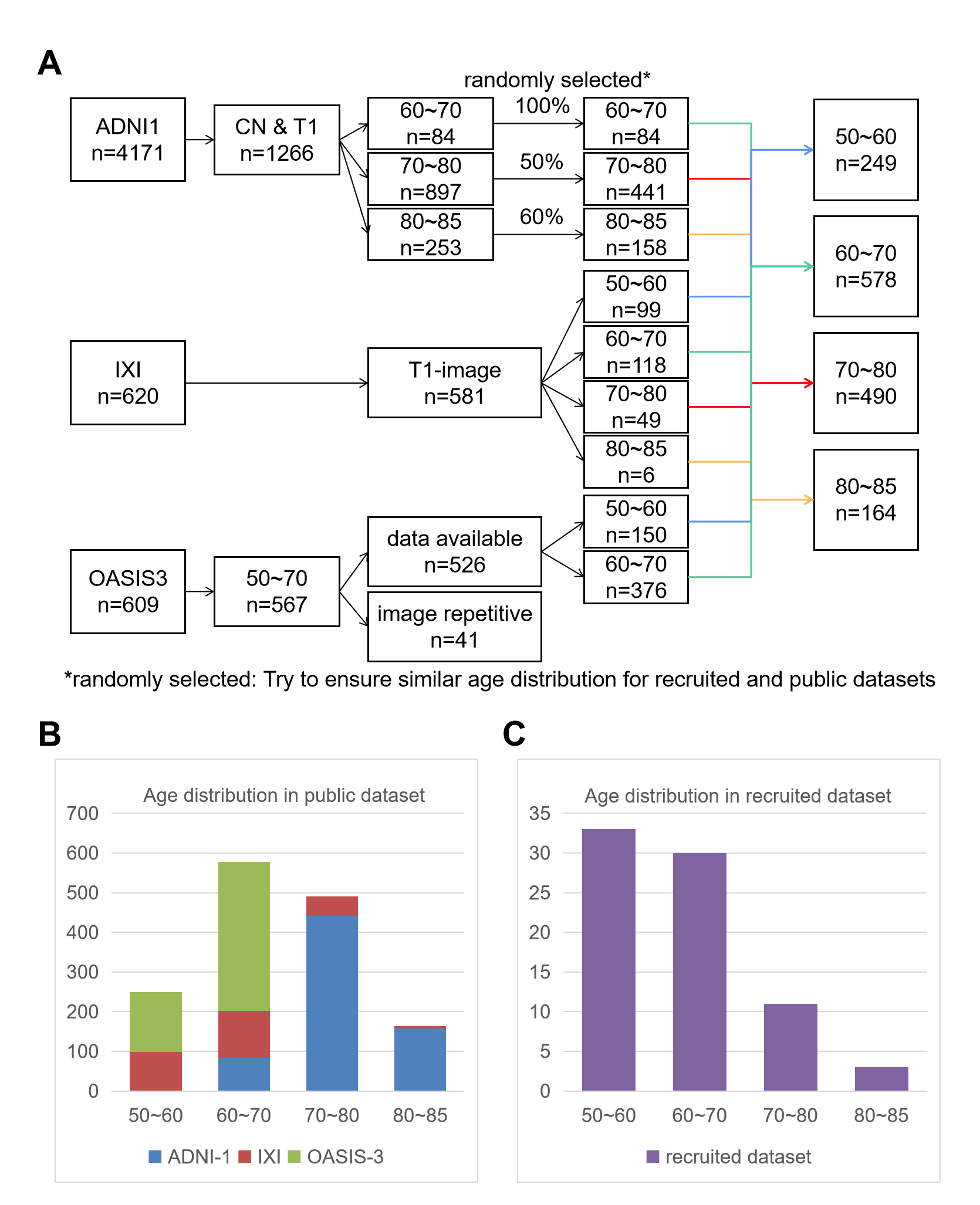

Supplement: Supplementary file 2 — Figure S2 The public dataset selection strategies and dataset's age distribution. (a) The collected criteria for ADNI, IXI, and OASIS‐3 database, (b) age distribution in public dataset, (c) age distribution in our recruited dataset. HC, health control. [file HBM-43-1640-s003.tif]

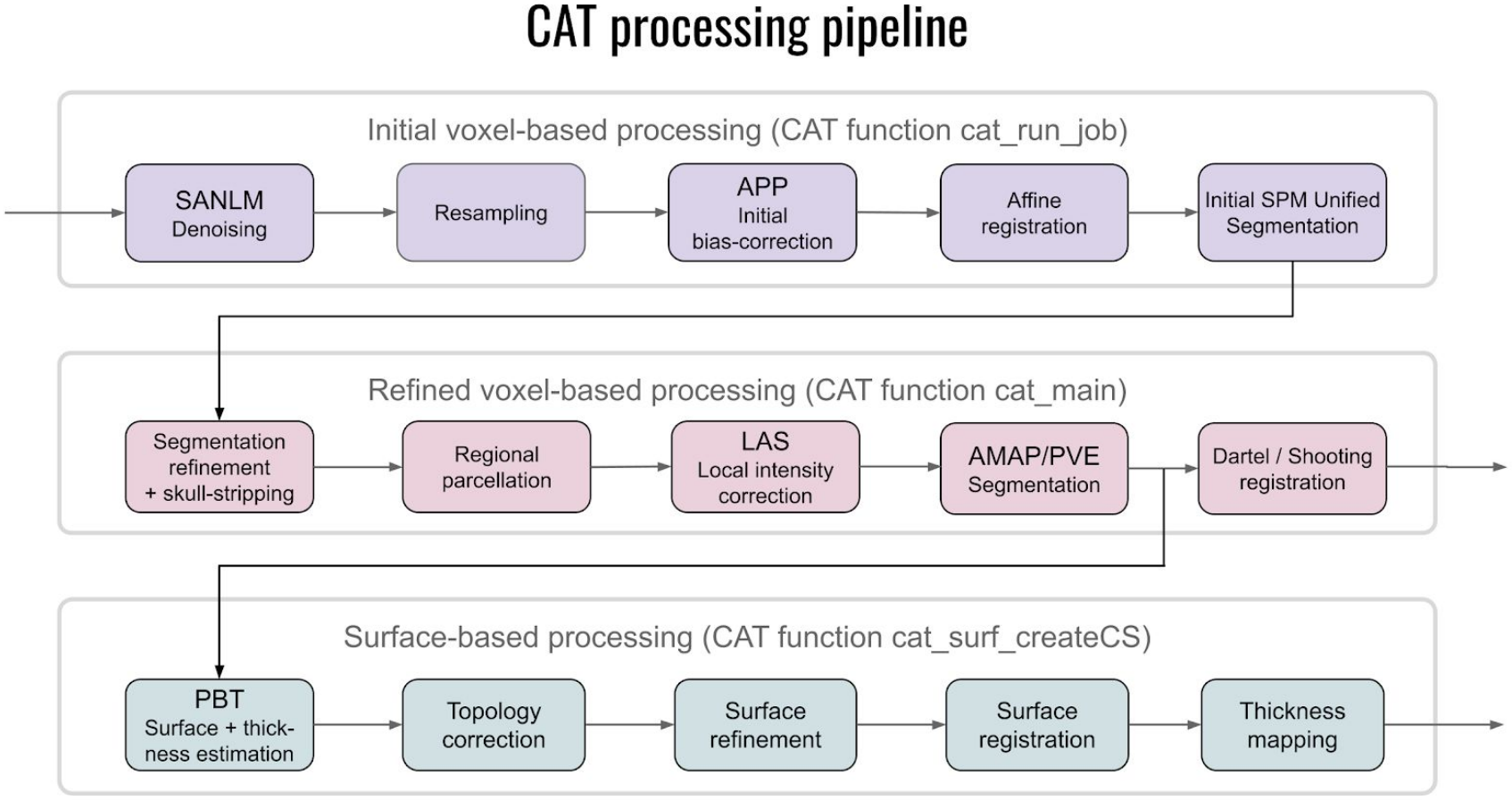

Supplement: Supplementary file 3 — Figure S3 CAT processing pipeline. [file HBM-43-1640-s001.tif]
