# Supplementary material for: Deep transfer learning of structural magnetic resonance imaging fused with blood parameters improves brain age prediction
Source: Hum Brain Mapp. 2021 Dec 16;43(5):1640–56. doi: 10.1002/hbm.25748 (PMC8886664; doi:10.1002/hbm.25748)
Supplement: Supplementary file 4 — Table S1 Concentrations of blood biochemical indicators and dementia biomarkers for each group and group comparisons. Table S2. Relationships between age and blood indicator concentrations. Table S3. The 45 anatomical brain regions ranked by the importance in the age prediction model. Table S4. Correlations between brain anatomical features and age‐related blood parameters. [file HBM-43-1640-s004.docx]

**Supplementary Figure 1.** Detailed Graphical representation of the network architecture.


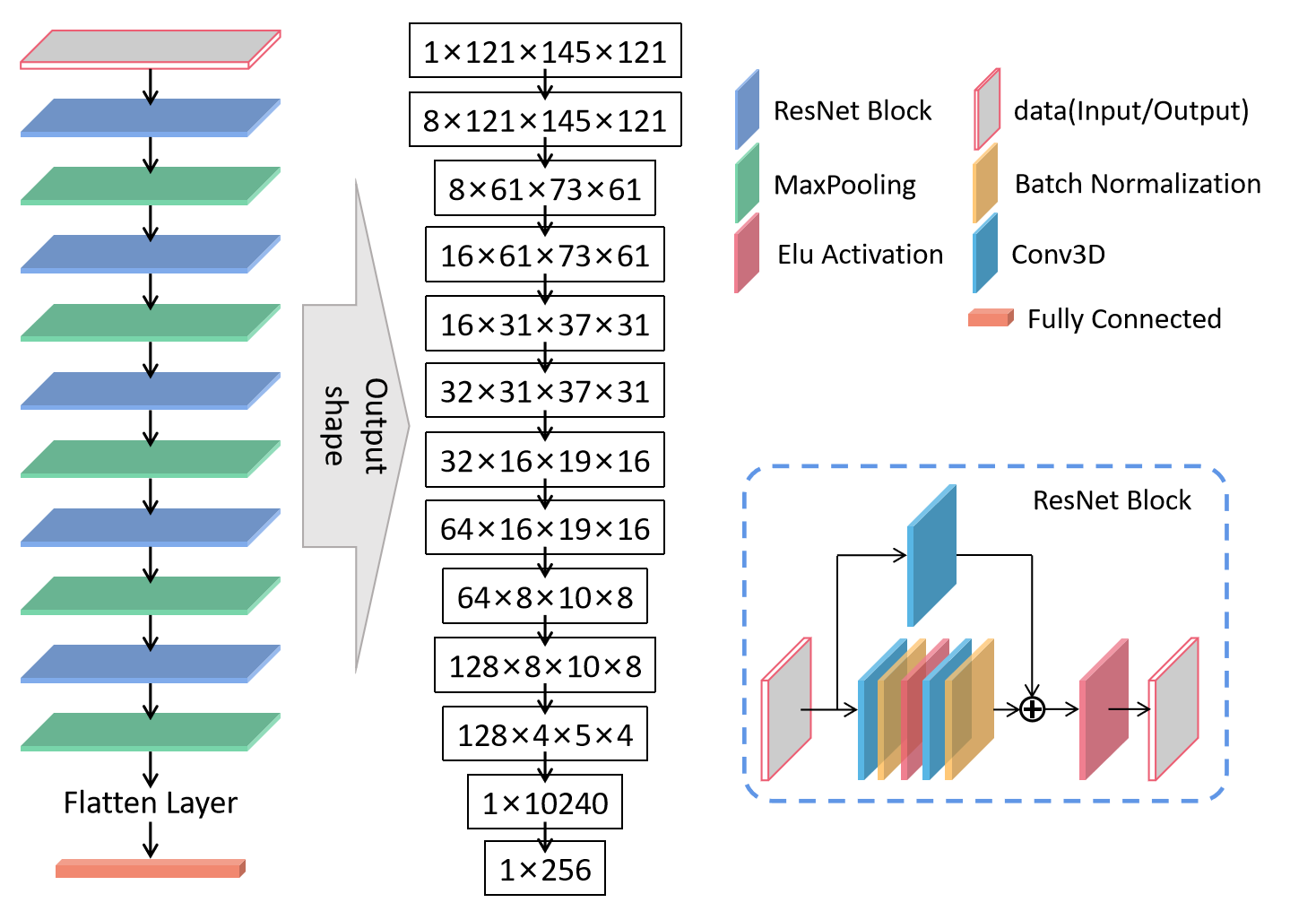


**Supplementary Figure 2.** The Public dataset selection strategies and dataset’s age distribution. (A) The collected criteria for ADNI, IXI and OASIS-3 database, (B) Age distribution in public dataset, (C) Age distribution in our recruited dataset. HC: Health Control.

**
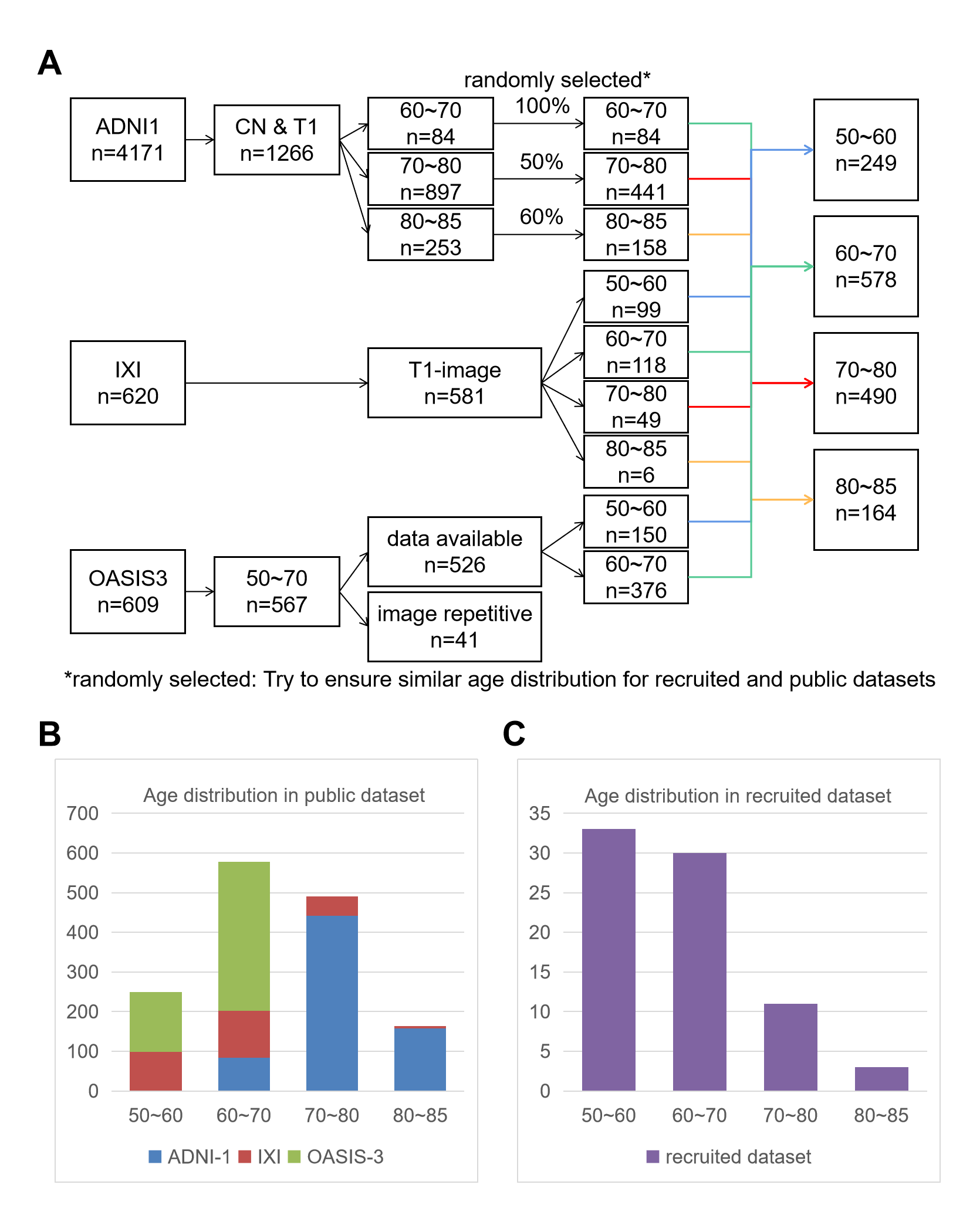
**

**Supplementary Figure 3.** The Pipeline of Voxel-based morphometry (VBM) in computational anatomy toolbox 12 (CAT12).


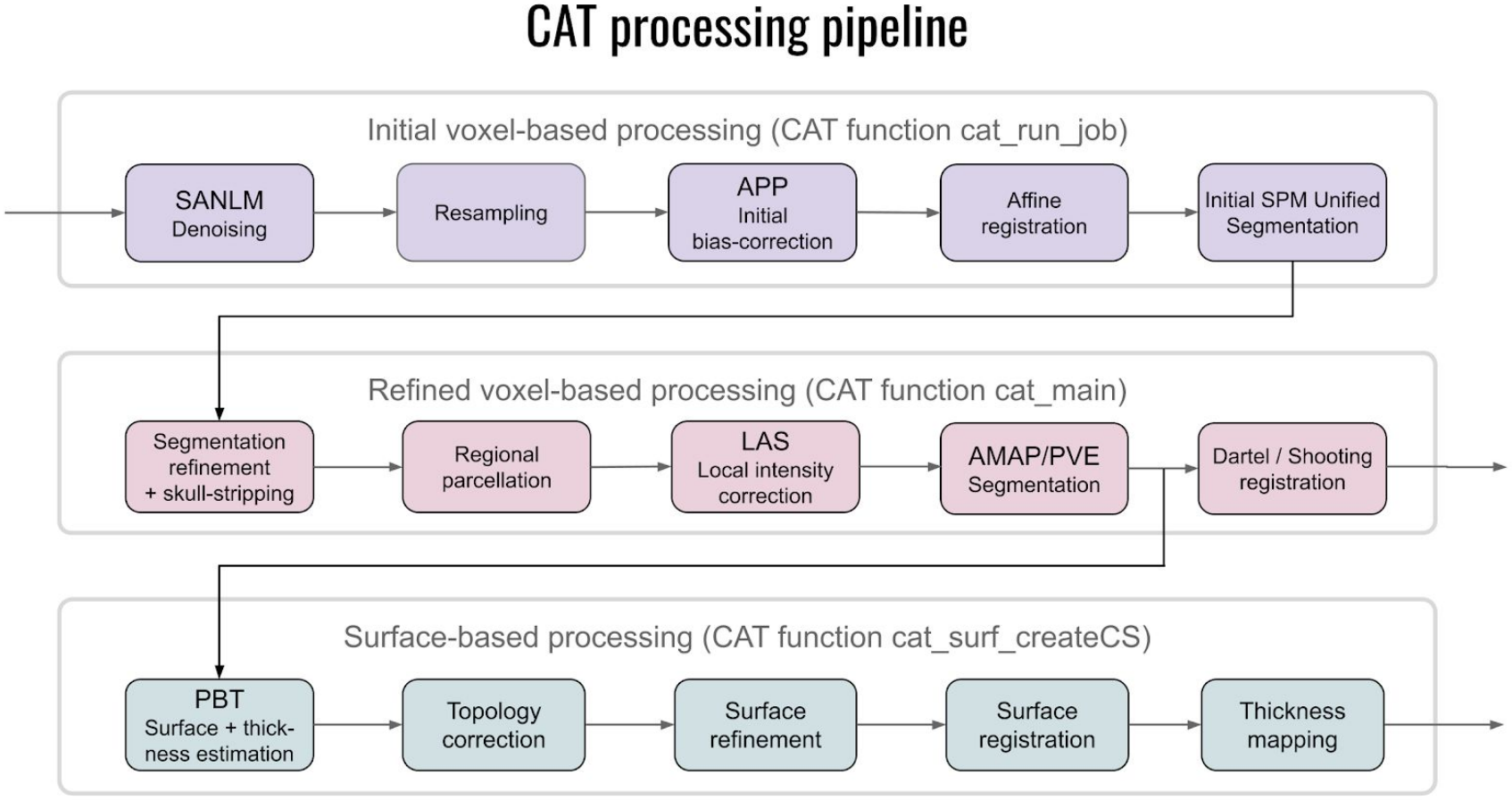


**Supplementary Table1.** Concentrations of blood biochemical indicators and dementia biomarkers for each group and group comparisons.

| Age (years) | Normality Test | 50~60 | 60~70 | Over 70 | Group comparison | post hoc |
| --- | --- | --- | --- | --- | --- | --- |
| GLU (mmol/L) | ***p<0.001 | 5.40 (4.90-6.60) | 5.40 (4.60-7.90) | 5.80 (5.10-13.70) | F= 4.206,  *p* < 0.05^a^ | 50~60 vs 60~70, *p* >0.999  50~60 vs Over 70, **p* < 0.05  60~70 vs Over 70, **p* < 0.05 |
| TG (mmol/L) | ***p<0.001 | 1.46 (0.54-13.94) | 1.26 (0.62-2.90) | 1.27 (0.83-3.94) | F= 0.150,  *p* = 0.962^b^,  *η^2^* = 0.008 | 50~60 vs 60~70, *p* = 0.816  50~60 vs Over 70, *p* = 0.580  60~70 vs Over 70, *p* = 0.498 |
| TC (mmol/L) | **p=0.005 | 4.56 (3.64-7.96) | 4.78 (2.49-7.00) | 5.14 (3.50-8.11) | F= 0.311,  *p* = 0.870^b^,  *η^2^* = 0.017 | 50~60 vs 60~70, *p* = 0.667  50~60 vs Over 70, *p* = 0.977  60~70 vs Over 70, *p* = 0.644 |
| ApoA1 (g/L) | *p=0.022 | 0.88 (0.70-1.52) | 0.97 (0.63-1.50) | 0.98 (0.73-1.45) | F= 2.869,  *p* < 0.05^b^,  *η^2^* = 0.137 | 50~60 vs 60~70, *p* = 0.073  50~60 vs Over 70, *p* = 0.080  60~70 vs Over 70, *p* = 0.659 |
| ApoB (g/L) | ***p<0.001 | 0.59 (0.44-1.07) | 0.59 (0.31-0.91) | 0.64 (0.39-1.06) | F= 0.510,  *p* = 0.729^b^,  *η^2^* = 0.028 | 50~60 vs 60~70, *p* = 0.499  50~60 vs Over 70, *p* = 0.594  60~70 vs Over 70, *p* = 0.267 |
| HCY (μmol/L) | ***p<0.001 | 9.20 (5.20-26.90) | 11.20 (7.20-23.90) | 12.15 (8.60-32.20) | F = 15.649  *p* < 0.001^b^  *η^2^* = 0.523 | 50~60 vs 60~70, ****p* <0.001  50~60 vs Over 70, ***p* < 0.01  60~70 vs Over 70, *p* =0.315 |
| NFL (pg/mL) | ***p<0.001 | 76.96 (43.43-140.77) | 94.08 (41.80-533.93) | 135.27 (90.57-380.13) | F = 6.271  *p* < 0.001^b^  *η^2^* = 0.272 | 50~60 vs 60~70, ****p* <0.001  50~60 vs Over 70, ***p* < 0.01  60~70 vs Over 70, ***p <*0.01 |
| TREM2 (pg/mL) | ***p<0.001 | 26.20 (26.20-633.44) | 26.20 (26.20-4698.44) | 26.20 (6.30-827.87) | F = 0.973  *p* = 0.428^b^  *η^2^* = 0.051 | 50~60 vs 60~70, *p* = 0.381  50~60 vs Over 70, *p* =0.293  60~70 vs Over 70, *p* = 0.086 |
| Aβ40 (pg/mL) | p=0.077 | 260.32 (150.79-403.50) | 278.44 (196.03-538.90) | 310.88 (191.22-429.74) | F = 0.208  *p* = 0.092^b^  *η^2^* = 0.104 | 50~60 vs 60~70, **p* < 0.05  50~60 vs Over 70, *p* = 0.092  60~70 vs Over 70, *p* = 0.252 |
| Aβ42 (pg/mL) | p=0.200 | 10.76 (6.02-15.07) | 11.91 (2.85-17.29) | 10.30 (8.21-15.42) | F = 1.800  *p* = 0.138^b^  *η^2^* = 0.092 | 50~60 vs 60~70, *p* =0.464  50~60 vs Over 70, *p* = 0.665  60~70 vs Over 70, *p* = 0.681 |
| T-tau (pg/mL) | p=0.200 | 2.60 (1.31-4.14) | 2.77 (1.14-5.68) | 2.57 (1.69-3.48) | F = 0.655  *p* = 0.625^b^  *η^2^* = 0.035 | 50~60 vs 60~70, *p* =0.827  50~60 vs Over 70, *p* = 0.513  60~70 vs Over 70, *p* = 0.458 |
| TIMP1 | *p=0.021 | 47.88 (14.12-128.25) | 51.30 (3.67-211.88) | 79.31 (14.32-164.61) | F = 2.894  *p* = 0.062^a^ | 50~60 vs 60~70, *p* =0.725  50~60 vs Over 70, *p* = 0.415  60~70 vs Over 70, *p* = 0.057 |
| VLDLR | ***p<0.001 | 19.42 (11.06-400.43) | 16.95 (8.53-335.40) | 17.72 (12.55-24.92) | F = 1.747  *p* = 0.149^b^  *η^2^* = 0.088 | 50~60 vs 60~70, *p* =0.445  50~60 vs Over 70, *p* = 0.902  60~70 vs Over 70, *p* = 0.495 |

The values were given as median (range).

a, one-way ANOVA; b, ANCOVA.

**p* < 0.05, ***p* < 0.01 and ****p*<0.001 were considered as statistically significant.

GLU, blood glucose; TG, triglyceride; TC, total cholesterol; ApoA1, apolipoprotein A1; ApoB, apolipoprotein B; HCY, homocysteine; NFL, Neurofilament light chain; TREM2, Triggering Receptor Expressed on Myeloid cells 2; Aβ, amyloid-beta; TIMP1, Tissue Inhibitor of Metalloproteinases 1; VLDLR, Very Low Density Lipoprotein Receptor.

**Supplementary Table2.** Relationships between age and blood parameters.

|  | Age | GLU | TG | TC | ApoA1 | ApoB | HCY | NFL | TREM2 | Aβ40 | Aβ42 | T-tau | Aβ42/40 | Aβ42/T-tau | TIMP1 | VRDLR |
| --- | --- | --- | --- | --- | --- | --- | --- | --- | --- | --- | --- | --- | --- | --- | --- | --- |
| Age | 1 | 0.053 | -0.055 | 0.118 | 0.318^**^ | 0.113 | 0.194 | 0.576^***^ | -0.209 | 0.363^**^ | -0.007 | 0.044 | -0.304^**^ | -0.246^*^ | 0.176 | -0.285^*^ |
| GLU |  | 1 | 0.133 | 0.256^*^ | -0.139 | 0.094 | 0.289^*^ | 0.128 | -0.061 | 0.004 | 0.031 | 0.184 | -0.019 | -0.138 | -0.073 | -0.018 |
| TG |  |  | 1 | 0.162 | -0.236^*^ | 0.126 | 0.015 | -0.014 | -0.097 | -0.076 | 0.089 | -0.150 | 0.135 | 0.255^*^ | 0.095 | 0.029 |
| TC |  |  |  | 1 | 0.189 | 0.818^***^ | 0.138 | 0.103 | -0.018 | -0.072 | -0.043 | -0.127 | -0.012 | 0.082 | 0.041 | 0.132 |
| ApoA1 |  |  |  |  | 1 | 0.037 | -0.098 | 0.083 | 0.051 | -0.114 | -0.025 | -0.020 | 0.051 | 0.059 | 0.040 | -0.070 |
| ApoB |  |  |  |  |  | 1 | 0.143 | 0.102 | -0.116 | -0.039 | -0.026 | -0.160 | -0.037 | 0.085 | 0.030 | 0.103 |
| HCY |  |  |  |  |  |  | 1 | 0.153 | 0.118 | -0.088 | -0.129 | -0.038 | 0.014 | 0.049 | 0.060 | -0.145 |
| NFL |  |  |  |  |  |  |  | 1 | -0.054 | 0.233^*^ | 0.112 | 0.066 | -0.110 | -0.155 | 0.088 | -0.036 |
| TREM2 |  |  |  |  |  |  |  |  | 1 | 0.061 | -0.362^**^ | 0.197 | -0.385^**^ | -0.276^*^ | 0.012 | -0.002 |
| Aβ40 |  |  |  |  |  |  |  |  |  | 1 | 0.264^*^ | 0.320^**^ | -0.586^***^ | -0.590^***^ | 0.288^*^ | 0.164 |
| Aβ42 |  |  |  |  |  |  |  |  |  |  | 1 | 0.290^*^ | 0.584^***^ | 0.171 | -0.109 | 0.047 |
| T-tau |  |  |  |  |  |  |  |  |  |  |  | 1 | -0.006 | -0.686^***^ | -0.180 | 0.169 |
| Aβ42/40 |  |  |  |  |  |  |  |  |  |  |  |  | 1 | 0.632^***^ | -0.299^**^ | -0.114 |
| Aβ42/T-tau |  |  |  |  |  |  |  |  |  |  |  |  |  | 1 | -0.105 | -0.079 |
| TIMP1 |  |  |  |  |  |  |  |  |  |  |  |  |  |  | 1 | 0.042 |
| VLDLR |  |  |  |  |  |  |  |  |  |  |  |  |  |  |  | 1 |

Age & ApoA1: ***p* = 0.005; Age & NFL: ****p* < 0.001; Age & Aβ40: ***p* = 0.001; Age & Aβ42/40: ***p* = 0.008; Age & Aβ42/T-tau: **p* = 0.031; Age & VLDLR: **p* = 0.012; GLU & TC: **p* = 0.025; GLU & HCY: **p* = 0.011; TG & ApoA1: **p* = 0.038; TG & Aβ42/T-tau: **p* = 0.025; TC & ApoB: ****p* < 0.001; NFL & Aβ40: **p* = 0.041; TREM2 & Aβ42: ***p* = 0.001; TREM2 & Aβ42/40: ***p* = 0.001; TREM2 & Aβ42/T-tau: **p* = 0.015; Aβ40 & Aβ42: **p* = 0.020; Aβ40 & T-tau: ***p* = 0.005; Aβ40 & Aβ42/40: ****p* < 0.001; Aβ40 & Aβ42/T-tau: ****p* < 0.001; Aβ40 & TIMP1: **p* = 0.011; Aβ42 & T-tau: **p* = 0.011; Aβ42 & Aβ42/40: ****p* < 0.001; T-tau & Aβ42/T-tau: ****p* < 0.001; Aβ42/40 & Aβ42/T-tau: ****p* < 0.001; Aβ42/40 & TIMP1: ***p* = 0.009.

**Supplementary Table 3.** The 45 anatomical brain regions ranked by the importance in the age prediction model.

| **Brain region** | **Size (voxels)** | **Importance (normalized)** | **Brain region** | **Size (voxels)** | **Importance (normalized)** |
| --- | --- | --- | --- | --- | --- |
| Amygdala | 948 | 1.00 | Superior Frontal Orbital | 4411 | 0.30 |
| Pallidum | 1291 | 0.99 | Calcarine | 9068 | 0.27 |
| Olfactory | 1245 | 0.93 | Inferior Frontal Triangular | 9649 | 0.25 |
| Putamen | 3984 | 0.92 | Anterior Cingulum | 5941 | 0.24 |
| Hippocampus | 4081 | 0.89 | Precuneus | 15510 | 0.23 |
| Thalamus | 4374 | 0.87 | Medial Frontal Orbital | 3276 | 0.23 |
| Fusiform | 11463 | 0.85 | Middle Cingulum | 9319 | 0.22 |
| Parahippocampus | 5387 | 0.85 | Central Paracentral Lobule | 5098 | 0.14 |
| Insula | 7377 | 0.77 | Supramarginal | 6420 | 0.12 |
| Lingual | 9881 | 0.67 | Superior Motor | 9869 | 0.12 |
| Caudate | 4354 | 0.64 | Middle Frontal Orbital | 4239 | 0.12 |
| Superior Temporal Pole | 6586 | 0.58 | Angular | 7024 | 0.11 |
| Inferior Temporal | 14160 | 0.56 | Cuneus | 7045 | 0.10 |
| Heschl | 1055 | 0.55 | Middle Occipital | 12213 | 0.09 |
| Rectus | 3264 | 0.53 | Inferior Parietal | 9121 | 0.08 |
| Median Cingulum | 1490 | 0.46 | Precentral | 16007 | 0.08 |
| Inferior Occipital | 4369 | 0.46 | Postcentral | 16808 | 0.07 |
| Rolandic Operculum | 5415 | 0.43 | Superior Parietal | 10737 | 0.06 |
| Inferior Frontal Orbital | 7610 | 0.43 | Superior Occipital | 6562 | 0.04 |
| Middle Temporal Pole | 4341 | 0.42 | Middle Frontal | 22474 | 0.04 |
| Superior Temporal | 13685 | 0.41 | Superior Frontal | 16235 | 0.01 |
| Inferior Frontal Operculum | 4972 | 0.40 | Superior Medial Frontal | 10853 | 0 |
| Middle Temporal | 20245 | 0.36 |  |  |  |

**Supplementary Table 4**. Correlations between brain anatomical features and age-related blood parameters.

|  | Amygdala Volume | Pallidum Volume | Putamen Volume | Hippocampus Volume | Olfactory Surface area | Olfactory GMV | Parahippocampus Surface area | Parahippocampus GMV | ApoA1 | VRDLR | NFL | Aβ1-40 |
| --- | --- | --- | --- | --- | --- | --- | --- | --- | --- | --- | --- | --- |
| Amygdala Volume | 1 | 0.465*** | 0.551*** | 0.62*** | 0.323** | 0.485*** | 0.393*** | 0.369*** | -0.024 | 0.137 | -0.173 | -0.115 |
| Pallidum Volume |  | 1 | 0.528*** | 0.652*** | -0.008 | 0.25* | 0.521*** | 0.329** | -0.295** | 0.089 | -0.348** | -0.154 |
| Putamen Volume |  |  | 1 | 0.547*** | 0.462*** | 0.452*** | 0.304** | 0.416*** | -0.172 | 0.106 | -0.327** | -0.129 |
| Hippocampus Volume |  |  |  | 1 | 0.253* | 0.444*** | 0.46*** | 0.451*** | -0.135 | 0.217 | -0.425*** | -0.153 |
| Olfactory Surface area |  |  |  |  | 1 | 0.717*** | -0.032 | 0.146 | -0.103 | 0.151 | -0.206 | -0.134 |
| Olfactory GMV |  |  |  |  |  | 1 | 0.152 | 0.225*** | -0.121 | 0.145 | -0.19 | -0.143 |
| Parahippocampus Surface area |  |  |  |  |  |  | 1 | 0.762*** | -0.193 | 0.195 | -0.178 | -0.099 |
| Parahippocampus GMV |  |  |  |  |  |  |  | 1 | -0.122 | 0.3** | -0.234* | -0.072 |
| ApoA1 |  |  |  |  |  |  |  |  | 1 | 0.033 | 0.108 | -0.107 |
| VRDLR |  |  |  |  |  |  |  |  |  | 1 | -0.142 | -0.011 |
| NFL |  |  |  |  |  |  |  |  |  |  | 1 | 0.451*** |
| Aβ1-40 |  |  |  |  |  |  |  |  |  |  |  | 1 |

Amygdala Volume & Pallidum Volume: ****p* < 0.001; Amygdala Volume & Putamen Volume: ****p* < 0.001; Amygdala Volume & Hippocampus Volume: ****p* < 0.001; Amygdala Volume & Olfactory Surface area: ***p* = 0.004; Amygdala Volume & Olfactory GMV: ****p* < 0.001; Amygdala Volume & Parahippocampus Surface area: ****p* < 0.001; Amygdala Volume & Parahippocampus GMV: ****p* = 0.001;

Pallidum Volume & Putamen Volume: ****p* < 0.001; Pallidum Volume & Hippocampus Volume: ****p* < 0.001; Pallidum Volume & Olfactory GMV: **p* = 0.028; Pallidum Volume & Parahippocampus GMV: ***p* = 0.003; Pallidum Volume & Parahippocampus Surface area: ****p* < 0.001; Pallidum Volume & ApoA1: ***p* = 0.009; Pallidum Volume & NFL: **p* = 0.002;

Putamen Volume & Hippocampus Volume: ****p* < 0.001; Putamen Volume & Olfactory Surface area: ****p* < 0.001; Putamen Volume & Olfactory GMV: ****p* < 0.001; Putamen Volume & Parahippocampus Surface area: ***p* = 0.007; Putamen Volume & Parahippocampus GMV: ****p* < 0.001; Putamen Volume & NFL: ***p* = 0.004;

Hippocampus Volume & Olfactory Surface area: **p* = 0.026; Hippocampus Volume & Olfactory GMV: *** *p* < 0.001; Hippocampus Volume & Parahippocampus Surface area: *** *p* < 0.001; Hippocampus Volume & Parahippocampus GMV: *** *p* < 0.001; Hippocampus Volume & NFL: *** *p* < 0.001;

Olfactory Surface area & Olfactory GMV: *** *p* < 0.001;

Olfactory GMV & Parahippocampus GMV: * *p* = 0.049;

Parahippocampus Surface area & parahippocampus GMV: *** *p* < 0.001;

Parahippocampus GMV & VRDLR: ** *p* = 0.008; Parahippocampus GMV & NFL: * *p* = 0.041;

NFL & Aβ1-40: *** *p* < 0.001.

GMV = Gray Matter Volume.
